# Generating human primordial germ cell-like cells from pluripotent stem cells: a scoping review of *in vitro* methods

**DOI:** 10.1093/hropen/hoaf056

**Published:** 2025-09-04

**Authors:** Madalena Vaz Santos, Ilse J de Bruin, Nina Dartée, Mathangi Lakshmipathi, Geert Hamer, Callista L Mulder, Willy M Baarends, Ans M M Van Pelt, Susana M Chuva De Sousa Lopes

**Affiliations:** Amsterdam Reproduction and Development Research Institute, Amsterdam, The Netherlands; Reproductive Biology Laboratory, Center for Reproductive Medicine, Amsterdam UMC location University of Amsterdam, Amsterdam, The Netherlands; Department of Developmental Biology, Erasmus MC, Rotterdam, The Netherlands; Department of Developmental Biology, Erasmus MC, Rotterdam, The Netherlands; Amsterdam Reproduction and Development Research Institute, Amsterdam, The Netherlands; Reproductive Biology Laboratory, Center for Reproductive Medicine, Amsterdam UMC location University of Amsterdam, Amsterdam, The Netherlands; Amsterdam Reproduction and Development Research Institute, Amsterdam, The Netherlands; Reproductive Biology Laboratory, Center for Reproductive Medicine, Amsterdam UMC location University of Amsterdam, Amsterdam, The Netherlands; Amsterdam Reproduction and Development Research Institute, Amsterdam, The Netherlands; Reproductive Biology Laboratory, Center for Reproductive Medicine, Amsterdam UMC location University of Amsterdam, Amsterdam, The Netherlands; Department of Developmental Biology, Erasmus MC, Rotterdam, The Netherlands; Amsterdam Reproduction and Development Research Institute, Amsterdam, The Netherlands; Reproductive Biology Laboratory, Center for Reproductive Medicine, Amsterdam UMC location University of Amsterdam, Amsterdam, The Netherlands; Department of Anatomy and Embryology, Leiden University Medical Center, Leiden, The Netherlands; The Novo Nordisk Foundation Center for Stem Cell Medicine (reNEW), Leiden University Medical Center, Leiden, The Netherlands

**Keywords:** primordial germ cells, primordial germ cell-like cells, human, pluripotent stem cells, *in vitro* differentiation, protocol comparisons

## Abstract

**STUDY QUESTION:**

How do the methods and outcomes of established protocols to specify human primordial germ cell-like cells (hPGCLCs) *in vitro* compare to each other?

**SUMMARY ANSWER:**

All analyzed protocols were successful in generating hPGCLCs, and a few were able to induce further germ cell maturation.

**WHAT IS KNOWN ALREADY:**

There are a variety of protocols for generating hPGCLCs *in vitro*, each with its own advantages and disadvantages. To date no comparison has been made, hindering the practical application of *in vitro*-derived hPGCLCs in research and the advancement toward generating mature germ cells.

**STUDY DESIGN, SIZE, DURATION:**

For this scoping review, a systematic search for protocols was conducted in the databases Scopus and Web of Science, including publications since 2010. Search terms included human, differentiation/specification/induction, germ cell/oogonia/spermatogonia, and primordial.

**PARTICIPANTS/MATERIALS, SETTING, METHODS:**

Two separate authors performed the database search according to the inclusion/exclusion criteria. The data regarding the materials and methods as well as results of the included articles were extracted and organized based on protocol (cell type and culture system) and outcome.

**MAIN RESULTS AND THE ROLE OF CHANCE:**

A systematic search revealed 32 articles describing the generation of hPGCLCs. Of these, 24 articles contained an original hPGCLC differentiation protocol and 8 articles provided an extension of a previously published protocol. The extension protocols focused either on extending hPGCLC culture or maturing hPGCLCs further. The articles were compared regarding protocol methods and differentiation outcomes. The data showed that differentiation in 2D or 3D, in the presence of bone morphogenetic protein 4 (BMP4) (or retinoic acid), activated the WNT and NODAL signaling pathways to induce hPGCLCs. Further maturation (based on gene expression) was also achieved, depending on the inclusion of subsequent differentiation steps. The 2D culture systems showed high efficiency and scalability, while the 3D culture systems were more suitable for germ cell maturation purposes. Further improvements would include a deeper assessment of epigenetic and gene expression, functional analyses, and use of multiple cell lines to reflect protocol versatility.

**LIMITATIONS, REASONS FOR CAUTION:**

Only literature has been compared; no extensive experimental comparison or a meta-analysis was performed due to the heterogeneity in outcome measurements.

**WIDER IMPLICATIONS OF THE FINDINGS:**

This review offers a comparison of hPGCLC differentiation protocols and aims to aid researchers in selecting appropriate protocols and making informed modifications to the culture conditions to achieve efficient germ cell differentiation.

**STUDY FUNDING/COMPETING INTEREST(S):**

This study was funded by ZonMW (PSIDER 10250022120001) and by the Novo Nordisk Foundation (reNEW NNF21CC0073729). The authors declare no conflicts of interest.

**REGISTRATION NUMBER:**

N/A.

WHAT DOES THIS MEAN FOR PATIENTS? The formation of human reproductive cells, known as primordial germ cells, is the first step in the production of sperm or egg cells in our body. To successfully complete this step, primordial germ cells depend on specific signals, sent by neighboring cells or by other parts of the body. Although cells that resemble primordial germ cells have been differentiated from pluripotent stem cells in the lab with various efficiencies by different research groups, further maturation remains suboptimal and requires additional research. By comparing the culture conditions used in various studies, we obtained insights in the next steps to grow sperm and egg cells. This will ultimately help toward understanding the causes of human infertility and exploring future treatment options.

## Introduction

To better understand human gamete generation, researchers have focused on the development of *in vitro* models to mimic this process. The first step of *in vitro* gametogenesis (IVG) is the formation of human primordial germ cells (hPGCs), termed human primordial germ cell-like cells (hPGCLCs). Over the past decades, significant advances have been made in the development of protocols to generate hPGCLCs. An extensive comparison of these protocols could provide leads on how to achieve the next steps in IVG.

Currently, hPGCs are still not well characterized at a molecular level. For that reason, *in vivo* knowledge complemented with data from *in vitro* studies is fundamental to advance our understanding of early human germ cell developmental processes. It is estimated that the first hPGCs emerge in the human embryo around 12 days post-fertilization (Chen *et al.*, 2019; Popovic *et al.*, 2019). Although the site of origin of these cells remains elusive, a combination of *ex vivo* and *in vitro* datasets suggest a possible dual origin from both the posterior epiblast and the nascent amnion (Sasaki *et al.*, 2016; Chen *et al.*, 2019; Popovic *et al.*, 2019; Castillo-Venzor *et al.*, 2023). *In vitro* studies showed that the germ cell program is initially triggered in TFAP2A-positive progenitor cells, in response to bone morphogenetic protein (BMP) and WNT signaling. The activation of these signaling pathways induces expression of core hPGC markers TFAP2C, SOX17, and PRDM1 (or BLIMP1) (Irie *et al.*, 2015; Sasaki *et al.*, 2015; Kojima *et al.*, 2021). Simultaneously, these cells retain expression of pluripotency-associated markers such as POU5F1 (or OCT4) and NANOG (Chen *et al.*, 2019; Shono *et al.*, 2023). After the onset of organogenesis, around 4 weeks post fertilization (WPF), hPGCs migrate through the hindgut, toward the developing gonadal ridges. At this stage, hPGCs start to express migration-associated markers, including KIT, DMRT1, and CDH5 (Li *et al.*, 2017; Gomes Fernandes *et al.*, 2018; Chen *et al.*, 2019). A complex interplay of factors is thought to be involved in the migratory journey, such as alterations in the expression of cell adhesion molecules, interaction with peripheral autonomic nerve fibers, and response to signaling factors from the somatic environment, such as CXCL12 and stem cell factor (SCF) (Roelen and Chuva de Sousa Lopes, 2022). Ultimately, these factors are thought to guide the germ cells to the developing gonadal ridges, around 5 WPF (Mollgard *et al.*, 2010; Mamsen *et al.*, 2012; Heeren *et al.*, 2016). During the migration process, hPGCs undergo a reset of the epigenetic landscape. This process initiates after lineage specification, with a wave of genome-wide DNA demethylation that is mostly completed between 7 and 10 WPF in the gonadal hPGCs (Guo *et al.*, 2015; Tang *et al.*, 2015; Murase *et al.*, 2024). The epigenetic reprogramming comprises not only the conversion of 5-methylcytosine (5mC) into 5-hydroxymethylcytosine (5hmC) and downregulation of de novo methyltransferases DNMT3A and DNMT3B, but also alterations in the levels of the repressive histone marks, H3K27me3 and H3K9me2 (Tang *et al.*, 2015; Gruhn *et al.*, 2023). After migration, the gonadal niche is established and hPGCs interact with the somatic gonadal cells while expanding through consecutive mitotic divisions. Subsequently, the germ cells start to express markers associated with a more mature developmental state, such as DDX4 and DAZL (Anderson *et al.*, 2007; Hancock *et al.*, 2021). At this stage, sex differentiation is initiated based on the sex chromosomes (XX in females and XY in males) of the surrounding gonadal somatic cells. If germ cells are XX, their inactivated X chromosome is reactivated and the germ cells enter meiosis, and differentiate to oocytes (Canovas *et al.*, 2017). If the surrounding gonadal somatic cells are XY, the germ cells, independently of their sex chromosomes, differentiate into gonocytes and are maintained in mitotic arrest in the seminiferous tubules until after birth (Nikolic *et al.*, 2016).

The difficult access to human embryos during gastrulation, when PGCs are specified, highlights the importance of using *in vitro* models to investigate this process. Many approaches have been pursued to differentiate hPGCLCs *in vitro*, starting from human pluripotent stem cells (hPSCs)—both human embryonic stem cells (hESCs) and human-induced pluripotent stem cells (hiPSCs). Initially, the generation of hPGCLCs was reported through spontaneous differentiation in embryoid bodies (EBs), without addition of growth factors (Clark *et al.*, 2004). However, advances in the generation of mouse PGCLCs (mPGCLCs) revealed the importance of BMP, activin A, and FGF signaling pathways (Hayashi *et al.*, 2011), and this knowledge was adapted to generate hPGCLCs. In recent years, -omics technologies, such as single-cell RNA sequencing (scRNA-seq) and epigenomics, have contributed to characterize the hPGCLCs extensively while allowing robust benchmarking against their *in vivo* counterparts.

There is currently a great diversity in differentiation protocols, starting materials, molecular characterization, and functional assessment. Here, we give an overview and comparison of original protocols for hPGCLC differentiation, which have been published in recent years. This scoping review provides a platform for the reader to efficiently review the established hPGCLC differentiation protocols, with an outline of their similarities and differences, and how these can affect the outcome.

## Methods

### Collection of articles

Research articles suitable for detailed protocol analyses were identified using the databases ‘Scopus’ and ‘Web of Science’. To limit the number of studies to a manageable scope, and with the rationale that most progress in hPGCLC differentiation protocols has been made in recent years, only papers published since 1 January 2010 were considered. Early access papers (pre-prints) were also included to capture the most recent advances. Review articles, book chapters, or errata were excluded. The initial searches were performed between 27 September 2023 and 11 October 2023. A final search to include recently published papers was performed on 17 January 2025. Additional articles were added after they had been found through snowballing using citations in identified articles. The resulting records identified through searching both databases were combined, after which duplicates were removed.

### Search strategies

The databases were searched for articles using the following combination of words in their title and/or abstract: human, differentiation, germ cell, and primordial. To include all relevant articles, variations of these words were also included, for example, ‘specification’ and ‘induction’ as a variation on ‘differentiation’. The two fixed search terms are as follows.

#### Search strategy used in Scopus

(TITLE-ABS (human*)) AND (TITLE-ABS (primordial*)) AND (TITLE-ABS (germ AND cell* OR germ-cell* OR oog? n* OR spermatog* OR gamet* OR pgc*)) AND (TITLE-ABS (generat* OR induc* OR differentiat* OR deriv* OR specif*)) AND PUBYEAR > 2009 AND (EXCLUDE(DOCTYPE, ‘re’) OR EXCLUDE (DOCTYPE, ‘ch’) OR EXCLUDE (DOCTYPE, ‘cp’) OR EXCLUDE (DOCTYPE, ‘er’))

#### Search strategy used in Web of Science

(TI = (human*) OR AB = (human*)) AND (TI = (generat* OR induc* OR differentiat* OR deriv* OR specif*) OR AB = (generat* OR induc* OR differentiat* OR deriv* OR specif*)) AND (TI = (primordial*) OR AB = (primordial*)) AND (TI=(germ cell* OR germ-cell* OR oog? n OR spermatog* OR gamet* OR PGC*))

### Inclusion/exclusion criteria

The identified articles were screened on title, abstract, and materials and methods. Articles were excluded based on the following reasons: non-research article, not English, non-human, germ-cell tumour, no iPSC/ESC starting material, no new protocol, exclusively *in vivo* or *ex vivo*, embryo model. The rationale of excluding based on ‘no iPSC/ESC starting material’ is that this scoping review compares protocols using hPSC to differentiate hPGCLC, hence excluding other cell types as starting material (such as umbilical cord cells). The ‘no new protocol’ exclusion criterium was applied when an article would refer to another published protocol in their methods, instead of developing their own original protocol. Articles describing maturation protocols starting from human primary germ cells or those that included the use of *in vivo* culturing/transplantation techniques were excluded. Additionally, articles using cultured germ cells or somatic cells directly isolated from gonads, without the addition of stem cell derived hPGCLCs, were excluded. During the searches, several articles were found where hPGCLCs spontaneously formed in embryo models. As these ‘embryo model’ protocols do not represent direct hPGCLCs, but constitute elaborate technical set-ups, they were excluded. Furthermore, differentiation protocols using genetic tools to overexpress or induce transcription factors to achieve differentiation were also excluded.

### Data analysis

Data were extracted from the articles and summarized in three tables. Each article was independently analyzed by two authors. Discrepancies were resolved through discussion to reach consensus, and the findings were summarized. The reported outcomes reflect only the data presented and discussed by the original authors in their respective articles. No additional analyses were performed on the retrieved data for this scoping review.

For each article, the protocol setup (as described in the original materials and methods section) was analyzed to determine the exact steps involved in each differentiation protocol. The analysis of differentiation outcomes (as described in the original results section) was assessed to define the basis on which their conclusions were reached. When an article tested variations of a protocol, only the variation(s) that yielded the best results were included. Furthermore, variations in (intermediate) timepoints were reported as ‘up to …’. In the protocol setup, the pre-treatment and intermediate steps of the protocols are taken into account, if applicable. Here, pre-treatment is considered any treatment (chemical or mechanical) applied to the cells before the start of differentiation to hPGCLCs. Intermediate states describe an intermediate cell state during stimulated differentiation.

In the differentiation outcomes, differentiation efficiency was quantified by FACS, while differentiation characteristics were assessed by gene expression data, immunofluorescence (IF) and epigenetic analyses. The marker genes were grouped based on the developmental stage of germ cells that they represent: pluripotency and pre-migratory, migratory and post-migratory, and meiotic and post-meiotic stage. Expression data were included only when initiated or upregulated as a result of the differentiation protocol, in comparison to the reference sample specific to the respective study. Additionally, we included cell functionality aspects reported in the studies, such as gene ontology enrichment, potential for maturation (with or without a somatic niche), localization of certain proteins, or activation of signaling pathways.

Finally, articles that were extensions to earlier published (and discussed) protocols were analyzed. Extensions included additional steps added to the original protocol to achieve either further maturation (differentiation of nascent hPGCLCs into a more advanced stage) or extended culture (proliferation culture or optimizing pre-treatment conditions). It is relevant to mention that the data presented in the tables is a synthesis of the details reported in the original studies.

## Results

### Literature search and study inclusion

**Figure 1.**
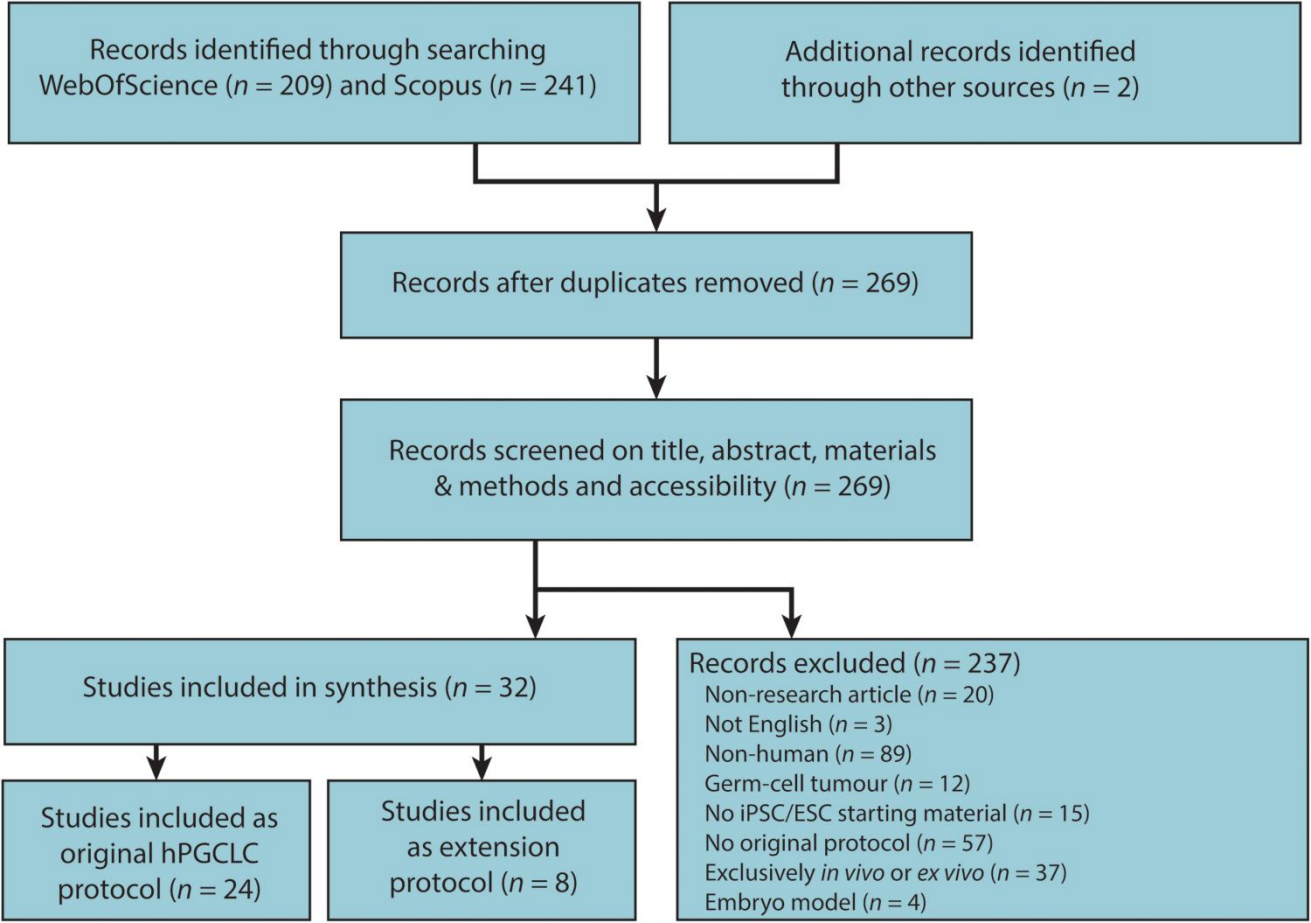
**Flow chart of the study selection.** Flow chart used for the selection of articles to include in this study based on PRISMA guidelines for scoping reviews, showing eligibility criteria for articles and the number of articles selected.

Through a systematic search based on keywords present in title and/or abstract, and after removal of duplicates, 269 articles were identified. Two articles were identified through snowballing citations in related articles. After screening the full text of the selected articles, 237 articles were excluded as they did not fulfill the eligibility criteria. In sum, 32 articles were included in our synthesis (Fig. 1).

### Data summary

**Figure 2.**
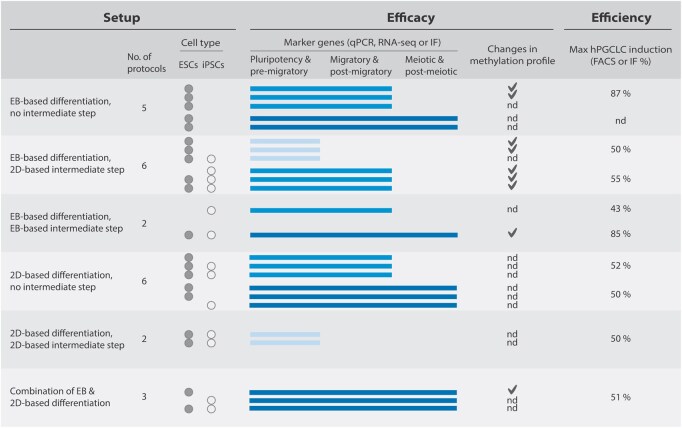
**Summary of setup, efficacy, and efficiency of the original protocols included in this study.** Summary of the experimental setup including the number of steps used, the type of pluripotent cells, the efficacy of differentiation to germ cells of different developmental stages, use of the methylation profile, and the differentiation efficiency as a percentage. The data on the original protocols are detailed on Tables 1 and 2. 2D: two-dimensional; EB: embryoid body; ESC: embryonic stem cell; IF: immunofluorescence; iPSC: induced pluripotent stem cell; hPGCLC: human primordial germ cell-like cell; nd: not determined; qPCR: quantitative polymerase chain reaction; RNA-seq: RNA sequencing.

The 32 studies were separated into two groups, based on whether the study presented an original hPGCLC differentiation protocol (24 studies) or an extension of a previously published protocol (8 studies). We identify commonalities and differences between the protocols and the types of (functional) assessment of the obtained hPGCLCs in both groups (Tables 1 and 2). Figure 2 provides the summarized data from the original hPGCLC protocols presented in Tables 1 and 2.

**Table 1.** Protocol setup of selected original studies.

Article	Steps	A. *Cell type*	B. *Cell line(s)*	*Pre-treatment*	*Intermediate*			*Differentiation*		
				C.	D. *Description*	E. *Induction/growth factors*	F. *Duration*	G. *Description*	H. *Induction/growth factors*	I. *Duration*
1. Alves-Lopes *et al*., 2023	EB-based differentiation, no intermediate step	ESC	WIS2 (XY, ESC), W24 (undefined, ESC), W15 (undefined, ESC)	ESC reset to naïve pluripotency (tt2iGöXAV and HENSM conditions)	None			1: EB in 96U-well (aRB27 basal medium^a^ + 0.25% PVA) and 2: Co-culture with human hindgut organoids in matrigel drops (aRB27 basal medium^a^)	1: 500 ng/ml BMP2, 100 ng/ml SCF, 50 ng/ml EGF, 10 ng/ml LIF, 10 μM iRock, 0.25% (v/v) PVA and 2: 100 ng/ml SCF, 25 ng/ml EGF, 10 μM iRock	1: 3–5 days and 2: up to 25 days
2. Duggal *et al.*, 2015		ESC	UGent11-6 (XX, ESC), UGent11-8 (XY, ESC), UGent11-4-ActA (XX, ESC), UGent12-3-ActA (XY, ESC)	Derivation in presence of ActA^b^	None			EB (knockout DMEM^c^ + 20% FBS + 1% P/S) + 2D attachment culture (0.1% gelatin + media 199 + 1% P/S + 1 mM L-glutamine + 0.3 mM sodium pyruvate + 0.8% human serum albumin)	D0–D6: 50 ng/ml BMP4, 20 ng/ml ActA; D7–D13: 10 ng/ml EGF, 1 µg/ml estradiol, 10 mIU/ml FSH, 0.5 IU/ml hCG	Up to 13 days
3. Irie *et al*., 2023		ESC	WIS2 (XY, ESC), Shef-6 (XX, ESC)	4i to maintain/induce naïve pluripotency	None			EB in 96U-well or aggrewell (aRB27 basal medium^a^ + 1% P/S)	D0–D3: 500 ng/ml BMP2, 100 ng/ml SCF, 50 ng/ml EGF, 10 μM iRock; D4–D11: 100 ng/ml ActA, 20 μM retinoic acid, 100 ng/ml SCF, 50 ng/ml EGF	Up to 11 days
4. Von Meyenn *et al.*, 2016		ESC	H9 (XX, ESC), WIBR3 OCT4-dPE-GFP (undefined, ESC)	EpiLC for primed pluripotency	None			EB in 96U-well (GK15 basal medium^d^)	500 ng/ml BMP4, 100 ng/ml SCF, 50 ng/ml EGF, 20 ng/ml LIF	12 days
5. Xuemei *et al.*, 2013		ESC	hES-8 (XX, ESC), hES-18 (XY, ESC)	None	None			EB (knockout DMEM^c^ + 20% FBS + 1% P/S)	10 μM all-trans retinoic acid	Up to 7 days
6. Irie *et al.*, 2015	EB-based differentiation, 2D-based intermediate step	ESC, iPSC	WIS2 (XY, ESC), WIBR3 (XX, ESC), LIS1 (XY, ESC), FX71.1 (FXS, iPSC), C1 (XX, iPSC)	4i to maintain/induce naïve pluripotency	2D culture for pre-induction (N2B27 basal medium^e^ + 1% KSR)	10 ng/ml bFGF, 1 ng/ml TGF-β1, or 20 ng/ml ActA, 10 μM iRock	48 h	EB in 96U-well (GK15 basal medium^d^ + 1% P/S)	500 ng/ml BMP4 or BMP2, 1 μg/ml LIF, 100 ng/ml SCF, 50 ng/ml EGF, 10 μM iRock	Up to 5 days
7. Kobayashi *et al.*, 2017		ESC	WIS2 (XY, ESC), WIBR3 (XX, ESC), LIS1 (XY, ESC), FX71.1 (FXS, iPSC), C1 (XX, iPSC), H9 (XX, ESC)	None	2D culture for mesoderm induction (aRB27 basal medium^a^)	100 ng/ml ActA, 3 μM CHIR99021, 10 μM iRock	12 h	EB (aRB27 basal medium^a^ + 2.5% PVA)	500 ng/ml BMP2, 100 ng/ml SCF, 50 ng/ml EGF, 10 ng/ml LIF, 10 μM iRock	Up to 5 days
8. Li *et al.*, 2019		ESC	H1 (XY, ESC)	None	2D culture for mesoderm induction (GK15 basal medium^d^)	40 ng/ml ActA, 3 μM CHIR99021, 10 μM iRocK	36–42 h	EB in 96U-well (aRB27 basal medium^a^)	500 ng/ml BMP4, 10 ng/ml LIF, 100 ng/ml SCF, 50 ng/ml EGF, 10 μM iRock, 50 μg/ml vitamin C	Up to 8 days
9. Sasaki *et al.*, 2015		iPSC	201B7 (XX, iPSC), 585A1 (XY, iPSC), 585B1 (XY, iPSC), BTAG 585B1-868 (XY, iPSC reporter for BLIMP1-tdTomato and TFAP2C-EGFP), 1383D2 (XY, iPSC), 1383D6 (XY, iPSC)	None	2D culture for mesoderm induction (GK15 basal medium^d^)	50 ng/ml ActA, 3 μM CHIR99021, 10 μM iRocK	42–48 h	EB in 96V-well (GK15 basal medium^d^)	1000 U/ml LIF, 200 ng/ml BMP4, 100 ng/ml SCF, 50 ng/ml EGF, 10 μM iRock	Up to 6 days
10. Sugawa *et al.*, 2015		ESC, iPSC	H9 (XX, ESC), HuES6 (XX, ESC), 393.2 (undefined, iPSC), SA8/25 (undefined, iPSC)	None	2D culture for mesoderm induction (N2B27 basal medium^e^)	5 ng/mL BMP4, 50 ng/mL ActA, 20 ng/mL bFGF, 10 μM iRock	48 h	EB in 96U-well (GK20 basal medium + 1% P/S)	100 ng/ml BMP4, 20 ng/ml LIF, 20 μM iRock	Up to 6 days
11. Wang *et al.*, 2019		ESC, iPSC	Fy-hES-3 (XY, ESC), Fy-hES-12 (XY, ESC), YiPS-1 (XY, iPSC), YiPS-2 (XY, iPSC)^f^	None	2D culture for mesoderm induction (GK15 basal medium^d^)	50 ng/ml ActA, 3 μM CHIR99021, 10 μM iRock	48 h	EB by drop seeding in 0.35% methylcellulose + floating culture (GK15 basal medium^d^)	500 ng/ml BMP4, 100 ng/ml SCF, 50 ng/ml EGF, 100 ng/ml LIF	Up to 8 days
12. Abdyyev *et al.*, 2020	EB-based differentiation, EB-based intermediate step	ESC, iPSC	hESMK05 (XX, ESC), 201B7 (XX, iPSC)	EB formation in aggrewell	EB formation (DK15^g^ medium)	50 ng/ml ActA, 10 μM iRock	36–42 h	1: EB in 96U-well (DK15^g^) and 2: Meiosis induction in embryoid bodies (DK3)	1: 100 ng/ml BMP4, 20 ng/ml LIF, 20 μM ROCKi and 2: 2 μM retinoic acid, 20 ng/ml LIF, 100 ng/ml EGF, 10 ng/ml SCF	1: 6 days and 2: 20 days
13. Mitsunaga *et al.*, 2017		iPSC	undefined	4i to maintain/induce naïve pluripotency	EB formation (4i medium^h^)	3 μM CHIR99021, 1 μM PD0325901, 2 μM BIRB796, 5 μM SP600125, 5 μM iRock	24 h	EB in floating culture 6-well (GK13 + 1% P/S + 50 μg/ml ascorbic acid)	10 μM iRock, 250–500 ng/ml BMP4, 100 ng/ml SCF, 50 ng/ml EGF, 1 μg/ml LIF	Up to 8 days
14. Cheng *et al.*, 2017	2D-based differentiation, no intermediate step	ESC	ZZU-hESC-2 (XY, ESC), ZZU-ESC-22 (XY, ESC)	Adaptation in MEF-conditioned medium	None			2D culture (DMEM/F12 + 20% KSR + 1% NEAA + 2 mM L-glutamine + 0.1 mM β-mercaptoethanol)	D0–D2: 8 μM CHIR99021; D3–D11: 2 μM retinoic acid	12 days
15. Duggal *et al.*, 2013		ESC	UGent11-5 (XX, ESC), UGent11-6 (XX, ESC), UGent11-7 (XY, ESC), UGent11-4-ActA (XX, ESC), UGent12-3-ActA (XY, ESC)	Derivation in presence of 20 ng/ml ActA	None			2D culture, low-oxygen condition (Knockout DMEM^c^ + 20% FBS + 1% P/S)	50 ng/ml BMP4	Up to 14 days
16. Esfahani *et al.*, 2024		ESC, iPSC	H9 (XX, ESC), H1 (XY, ESC), HES-3 (XX, ESC), 1196a (undefined, iPSC), eight NOA patient-derived cell lines (XY, iPSC)	Adherent culture as priming period (PSC medium + 10 μM iRock)	None			Adherent culture (mTeSR or Essential 6 media)	4% Geltrex	Up to 8 days
17. Overeem *et al.*, 2023		ESC, iPSC	H1 (XY, ESC), M54 (XY, iPSC), F99 (XX, iPSC), F31 (XX, iPSC), F20 (XX, iPSC), M72 (XY, iPSC), F198 (XX, iPSC), F198-POU5F1-GFP (XX, iPSC)	Adherent culture as priming period (PSC medium + 2% BMEx)	None			Adherent culture (aRB27 basal medium^a^ + 1% Mycozap)	D1/D2: 2% Geltrex/Cultrex, 10 ng/ml BMP4; D3–D5: 10 ng/ml BMP4, 10 ng/ml LIF, 50 ng/ml SCF, 50 ng/ml EGF	5 days
18. Panula *et al.*, 2011		iPSC	H9 (XX, ESC), HSF1 (XY, ESC), iPS(IMR90) (XX, iPSC), iHUF4 (XY, iPSC)	Adaptation in MEF-conditioned medium	None			2D culture (knockout DMEM^c^ + 20% fetal bovine serum)	50 ng/ml BMP4, BMP7 and BMP8b	Up to 14 days
19. Wongtrakoongate *et al.*, 2013		ESC	H7 (XX, ESC), Shef3 (XY, ESC)	None	None			2D culture on MMC-treated MF-1 MEFs (knockout DMEM^c^ + 20% knockout-serum replacement)	4 ng/ml bFGF, 10 μM all-trans retinoic acid	Up to 21 days
20. Jo *et al.*, 2022	2D-based differentiation, 2D-based intermediate step	ESC, iPSC	ESI017 (XX, ESC), PGP1 (XY, iPSC), WTC11 (XY, iPSC), MR30 (XX, iPSC)	Cells seeded in laminin-micropattern wells	2D culture for mesoderm induction	100 ng/ml ActA, 3 μM CHIR99021, 10 μM iRock	12 h	2D culture in laminin-micropattern wells, controlled colony size 100 μm (mTeSR1 medium + 10 μM iRock)	50 ng/ml BMP4	Up to 4 days
21. Vijayakumar *et al.*, 2023		ESC, iPSC	H1 (XY, ESC), H9 (XX, ESC), NANOS3-mCherry WIS1 (XX, ESC), SOX17-GFP H9 (XX, ESC), NANOG-2A-YFP H9 (XX, ESC), BJC1 (iPSC, undefined), BJC3 (iPSC, undefined), BIRc3 (iPSC, undefined)	None	2D culture for posterior epiblast induction (aRB27^a^ basal medium)	100 ng/ml ActA, 3 μM CHIR99021, 10 μM iRock	12 h	2D culture (aRB27 basal medium^a^ + 1% P/S)	D1: 40 ng/ml BMP4, 1 μM XAV939, 10 μM iRock; D2: 100 ng/ml SCF, 50 ng/ml EGF, 1 μM XAV939, 10 μM Y-27632; D3: 40 ng/ml BMP4, 100 ng/ml SCF, 50 ng/ml EGF, 1 μM XAV939, 10 μM iRock	3 days
22. Kiani *et al.*, 2023	Combination of EB and 2D-based differentiation	iPSC	Undefined	None	EB formation (DMEM/F12 + 20% KSR)	None	48 h	EB + 2D attachment culture (DMEM/F12 + 20% KSR + 1% P/S + 0.1 mM NEAA + 0.1 mM β-mercaptoethanol)	100 ng/ml BMP4, 0.1 μM retinoic acid	4 days
23. Lin *et al.*, 2014		ESC	H9 (XX, ESC), NTU1 (XX, ESC)	None	EB formation (ReproCELL medium or DMEM/F12 + 20% KSR)^i^	5 ng/ml bFGF	72 h	EB + 2D attachment culture (DMEM, 15% FBS + 0.5% antibiotics)	100 ng/ml BMP4, 50 ng/ml WNT3A	Up to 20 days
24. Yu *et al.*, 2023		ESC, iPSC	TAC153 (XX, iPSC), H9 (XX, ESC)	None	None			1 and 2: 2D culture (1) with aggregate detachment (2) (α-MEM + 5% KSR + 5% bovine follicular fluid + 1% P/S + 0.1% NEAA + 0.1 mM β-mercaptoethanol + 1% L-glutamine) and 3: Free floating aggregates (TCM 199 + 3 mg/ml BSA)	1 and 2: 50 ng/ml BMP4, 200 ng/ml LIF, 100 ng/ml SCF, 50 ng/ml EGF, 10 μM iRock; 3: 5 U/ml FSH, 10 U/ml hCG, 10 IU/ml PMSG, 10 ng/ml EGF, 1% ITS, 0.23 mM pyruvic acid	1: 10 days and 2: 5 days and 3: 10 days

a aRB27: advanced RPMI 1640, 1% B27 supplement, 0.1 mM NEAA, 2 mM L-glutamine.

b Derivation in presence of activin A described in Duggal *et al.* (2013) (protocol 15).

c Knockout DMEM: 2 mM L-glutamine, 1% NEAA, 0.1 mM β-mercaptoethanol.

d GK15: GMEM, 15% KSR, 1× NEAA, 2 mM L-glutamine, 1 mM sodium pyruvate, 0.1 mM β-mercaptoethanol.

e N2B27: 1:1 DMEM/F12, neurobasal medium, 0.5% N2 supplement, 1% B27 supplement, 2 mM MEM NEAA, 1% GlutaMax, 0.1 mM β-mercaptoethanol, 100 U/ml Penicillin, 100 μg/ml streptomycin.

f The iPSCs were derived from somatic testes cells.

g DK15: DMEM/F12, 15% KSR, 0.1 mM NEAA, 2 mM GlutaMax, 1 mM sodium pyruvate, 0.1 mM β-mercaptoethanol.

h 4i: 82% (vol/vol) Knockout DMEM, 16% (vol/vol) KSR, 1× penicillin-streptomycin-glutamine, 1× NEAA, 10.6 μg/ml bovine pancreas insulin, 16.3 ng/ml rhLIF, 6.5 ng/ml rhbFGF2, 1.6 ng/ml rhTGF-β1, 5 μM Y27632.

i Depends on cell line used.

iPSC: induced pluripotent stem cell; ESC: embryonic stem cell; MEF: mouse embryonic fibroblast; EpiLC: epiblast-like cell; hPGCLC: human primordial germ cell-like cell; EB: embryoid bodies; 2D: two dimensional; 3D: three dimensional; BMEx: basement membrane extract; ActA: activin A; BMP4: bone morphogenetic protein; bFGF: basic fibroblast growth factor; TGF-β1: transforming growth factor beta; SCF: stem cell factor; PVA: polyvinyl alcohol; FSH: follicle-stimulating hormone; hCG: human chorionic gonadotropin; WNT3A: Wnt family member 3A; PMSG: pregnant mare serum gonadotropin; ITS: insulin-transferrin-selenium; iRock: Rock inhibitor (Y-27632); CHIR99021: WNT activator; PD0325901: MEK inhibitor; BIRB796: p38 MAPK inhibitor; SP600125: JNK inhibitor; XAV939: TNKS/PARP inhibitor; MMC: mitomycin-C; P/S: penicillin-streptomycin; BSA: bovine serum albumin; FBS: fetal bovine serum; DMEM: Dulbecco’s modified eagle medium; GMEM: Glasgow’s eagle minimum essential medium; αMEM: minimum essential medium eagle alpha modification; KSR: knockout serum replacement; NEAA: non-essential amino acids.

**Table 2.** Differentiation outcomes of selected original studies.

Article	Steps	A. *FACS (ab/reporter/marker)*	*Markers expression*			E. *Sequencing*	F. *Epigenetic landscape*	G. *Remarks on functionality*	H. *Notes*
			B. *Pluripotency and pre-migratory*	C. *Migratory and post-migratory*	D. *Meiotic and post-meiotic*				
1. Alves-Lopes *et al.*, 2023	EB-based differentiation, no intermediate step	Up to 18.86% TNAP+ PDPN+; up to 15.16% TNAP+ NANOS3+	BLIMP1, SOX17, OCT4, NANOG, NANOS3 (bulk RNA-seq, IF); TFAP2C, DPPA3, PDPN (bulk RNA-seq)	DAZL (bulk RNA-seq, IF); DDX4, PIWIL2, KIT, CXCR4 (bulk RNA-seq)	None	Co-cultured resetting hPGCLCs (rhPGCLCs) progress from nascent rhPGCLCs toward 6–8 WPF hPGCs	5mC decrease; H3K9me2 decrease (in nascent rhPGCLCs vs neighboring somatic cells)	Colonization of the hindgut organoid; morphology of rhPGCLCs reminiscent of migratory hPGCs, being individually recognizable cells with cytoplasmic protrusions, 4i/pre-mesoderm derived hPGCLCs remained as tight clumps	EOMES is dispensable for rhPGCLC specification; rhPGCLCs mature faster (vs 4i/pre-mesoderm hPGCLC) when co-cultured with mouse somatic cells or hindgut organoids
2. Duggal *et al.*, 2015		None	OCT4, DPPA3^a^ (qPCR and IF)	DDX4 (qPCR, IF); KIT^a^, DAZL (qPCR)	SYCP3^a^ (qPCR)	None	None	None	None
3. Irie *et al.*, 2023		Up to 86.9% NANOS+ DMRT1+ cells at D8	BLIMP1, SOX17, NANOS3 (scRNA-seq, qPCR); OCT4 (IF)	DMRT1 (scRNA-seq, qPCR, IF); CXCR4 (scRNA-seq); CDH5 (qPCR)	None	hPGCLCs cluster together with migratory hPGCs	No 5mC or 5hmC alteration	GO analysis showed upregulation of PGC genes and cell migration, and downregulation of cell adhesion	Induction of DMRT1 (by doxycycline) activated DAZL expression and leads to DNA methylation changes
4. Von Meyenn *et al.*, 2016		Up to 25.9% KIT+	BLIMP1, OCT4, SOX17 (qPCR, IF); NANOG, NANOS3 (qPCR)	KIT (qPCR)	None	hPGCLCs cluster separately from ESCs and intermediate cells (bulk RNA-seq)	H3K9me2/3 downregulation	None	None
5. Xuemei *et al.*, 2013		None	None	DDX4 (qPCR)	SYCP3, GDF9, TEKT1 (qPCR)	None	None	None	None
6. Irie *et al.*, 2015	EB-based differentiation, 2D-based intermediate step	Up to 26.7% TNAP+ CD38+ at D4	OCT4, NANOS3, BLIMP1, TFAP2C, SOX17 (qPCR, IF, bulk RNA-seq); TNAP (qPCR, bulk RNA-seq); DPPA3 (qPCR)	KIT (qPCR, bulk RNA-seq)	None	hPGCLC hierarchically cluster with hPGCs, separately from soma and undifferentiated 4i ESCs and intermediate cells (bulk RNA-seq)	UHRF1, DNMT3A, DNMT3B downregulation, TET1, TET2 upregulation; 5mC decrease, 5hmC increase	GO analysis revealed enrichment of spermatogenesis genes in male hPGCLC and hPGC	None
7. Kobayashi *et al.*, 2017		Up to 38%, NANOS3+(reporter) TFAP2A+	OCT4, BLIMP1, SOX17, TFAP2C (qPCR, IF); NANOS3 (qPCR)	None	None	None	None	None	None
8. Li *et al.*, 2019		Up to 41.65% BLIMP1+(reporter) TNAP+; up to 55.23% ITGA6+ EpCAM+	BLIMP1, NANOS3, TFAP2C, OCT4, NANOG, TNAP, SOX17 (qPCR)	None	None	None	TET1, TET2 and TET3 upregulation; 5mC decrease, 5hmC increase	None	None
9. Sasaki *et al.*, 2015		Up to 30% BLIMP1+ TFAP2C+ (reporter); up to 50% ITGA6+ EpCAM+	BLIMP1, TFAP2C, SOX17 (qPCR, IF, bulk RNA-seq); POU5F1, NANOG, NANOS3, DPPA3^a^ (qPCR); reporters BTAG (IF)	CXCR4, KIT (bulk RNA-seq)	None	hPGCLCs, hPGCs and cynomolgus monkey PGCs hierarchically cluster together, separately from PSCs and intermediate cells (bulk RNA-seq)	H3K9me2 downregulation, H3K27me3^a^ upregulation; DNMT3B downregulation	GO analysis revealed upregulation of potential hPGCLC regulator genes	Gene expression profiles of germ cell-reporter cells and TNAP+NANOS+ cells are highly correlated
10. Sugawa *et al.*, 2015		Up to 20% TRA-1-81+ KIT+ at D4	BLIMP1, OCT4 (qPCR, IF, bulk RNA-seq); SOX17, TFAP2C (bulk RNA-seq); NANOG (qPCR); DPPA3 (qPCR, IF); PRDM14 (IF)	None	None	hPGCLC hierarchically cluster separately from PSC and intermediate states (microarray)	5mC decrease	GO analysis of hPGCLC DEGs revealed pattern of downregulation similar to mouse PGC specification	None
11. Wang *et al.*, 2019		Up to 39.8% ITGA6+ EpCAM+	BLIMP1, TFAP2C, SOX17 (IF, qPCR); NANOS3 (qPCR); OCT4 (IF)	DDX4 (qPCR)	None	None	DNMT3A, DNMT3B downregulation	None	8–10× higher hPGCLC yield compared to Sasaki (2015) for the same amount of medium consumption
12. Abdyyev *et al.*, 2020	EB-based differentiation, EB-based intermediate step	Up to 85% SSEA1+	1: [Pre-meiotic PGCLCs] OCT4, BLIMP1, NANOS3, SSEA1 (qPCR, IF); NANOG (qPCR); SOX17, DPPA3 (IF); 2: [Meiosis-induced PGCLCs] NANOS3 (qPCR); DPPA3 (IF)	1: DDX4 (qPCR, IF); downregulation KIT (qPCR); 2: DAZL (qPCR, IF); DDX4, PIWIL1, PIWIL4 (qPCR)	2: SYCP2^a^, SYCP3^a^, REC8 (qPCR); STRA8, SYCP3 (IF)	iPSCs, d6 EB-hPGCLCs and meiotic entry-like cells cluster separately; also cluster separately from previous reports (Yamashiro 2018) extension dataset (bulk RNA-seq)	1: DNMT3B downregulation	Studied the extracellular matrix expression profile, but concluded that the direct induction of derived EB-hPGCLCs with retinoic acid was not sufficient to lead to full meiosis	Presence of ECM and somatic male gonadal genes expression in meiotic-induced EBs suggests transdifferentiation to somatic lineage
13. Mitsunaga *et al.*, 2017		Up to 43.1% CD38+ at EB D8	BLIMP1, NANOS3, TFAP2C (qPCR, bulk RNA-seq); SOX17 (bulk RNA-seq); OCT4 (IF, bulk RNA-seq)	KIT (bulk RNA-seq); DPPA3 (qPCR)	None	hPGCLC hierarchically cluster with hPGCLC clusters from previous reports (Irie 2015, Sasaki 2015), separately from PSCs and human fetal germ cells (bulk RNA-seq)	None	Demonstrated that a 72 h period is sufficient for iPSC 4i capacitation for hPGCLC differentiation; GO analysis revealed enrichment of migratory genes in hPGCLC, paired with CXCR4 expression	None
14. Cheng *et al.*, 2017	2D-based differentiation, no intermediate step	Up to 26.9% KIT+; up to 2.95% CXCR4+; up to 24.7% DAZL+	BLIMP1, NANOS, TFAP2C (qPCR); OCT4 (IF)	DDX4 (qPCR); DAZL (IF)	SYCP3 (IF)	None	None	Relocation of beta-catenin into the nucleus during hPGCLC differentiation, suggesting activation of WNT pathway	None
15. Duggal *et al.*, 2013		None	SSEA1 (qPCR); OCT4 (IF)	DDX4 (qPCR, IF)	None	None	None	Presence of nuclear pSMAD2/3 and pSMAD1/5/8, confirms responsiveness to BMP and Activin/TG β signaling in hPGCLC differentiation potential	Initial inherent expression of DPPA3 and KIT in ActA-derived iPSC lines
16. Esfahani *et al.*, 2024		Up to 21% TFAP2C+ SOX17+	BLIMP1, OCT4, SOX17, TFAP2C, NANOG (scRNA-seq, IF); NANOS3 (scRNA-seq)	KLF4 (scRNA-seq, IF)	None	hPGCLCs at day 8 overlap with PGCs from a CS7 human gastrula (scRNA-seq)	None	GO analysis revealed upregulation of cell migration	Amnion-like cells induce hPGCLC specification, likely involving WNT and BMP signals
17. Overeem *et al.*, 2023		52.3% ITGA6+ EpCAM+	BLIMP1, SOX17, OCT4 (IF, scRNA-seq); PDPN, SOX17, TFAP2C (IF)	DDX4, DAZL (IF in co-cultures)	None	hPGCLC cluster with hPGC, with their transcriptome resembling Carnegie Stage 7 hPGC (scRNA-seq)	None	Maturation of hPGCLCs through co-culture with human fetal ovary cells	None
18. Panula *et al.*, 2011		Up to 5% DDX4+ (reporter)	BLIMP1, OCT4 (qPCR)	DDX4 (IF, WB); DAZL (WB)	DMC1 (qPCR)	None	None	None	None
19. Wongtrakoongate *et al.*, 2013		Up to 50% SSEA1+	None	DDX4 (qPCR)	ZP1 (qPCR)	None	None	None	None
20. Jo *et al.*, 2022	2D-based differentiation, 2D-based intermediate step	None (efficiency measured by IF quantification, up to 50% SOX17+ TFAP2C+ EOMES-)	BLIMP1, TFAP2C, SOX17, NANOG (IF, scRNA-seq); OCT4 (IF)	None	None	hPGCLC cluster with human Carnegie Stage 7 hPGC (scRNA-seq)	None	hPGCLC differentiation is controlled by exposure length/timing of BMP and NODAL induction, in response to WNT signaling; control of colony size dramatically improves hPGCLC differentiation efficiency	None
21. Vijayakumar *et al.*, 2023		Up to 28.3% NANOS+ (reporter)	BLIMP1, OCT4, SOX17, TFAP2C (qPCR, IF/FACS, scRNA-seq); NANOG, NANOS3 (IF, scRNA-seq)	None	None	hPGCLC cluster with human mitotic fetal germ cells (scRNA-seq)	None	None	Identified hPGCLC surface markers CXCR4+GARP-PDGFRa-, gene expression profile highly correlated with BLIMP1+SOX17+
22. Kiani *et al.*, 2023	Combination of EB and 2D-based differentiation	None	BLIMP1 (qPCR)	DDX4 (qPCR, IF); DAZL (qPCR); KIT (IF)	STRA8 (qPCR, IF)	None	None	None	None
23. Lin *et al.*, 2014		Up to 17% OCT4+ (reporter) at D15	OCT4, BLIMP1, NANOS3 (qPCR, IF); DPPA3 (IF)	DDX4^a^ (qPCR, IF)	SYCP3^a^ (qPCR, IF)	Expression profile of BLIMP-overexpressing ESCs correlates to early stage germ cells (microarray)	DNMT3A downregulation	Expression of BLIMP1 correlates with the suppression of SOX2, which is required for germline differentiation *in vitro*	None
24. Yu *et al.*, 2023		Up to 50.6% KIT+ at D10	BLIMP1, SOX17 (scRNA-seq, qPCR, IF); TFAP2C (scRNA-seq, qPCR); OCT4 (scRNA-seq, IF); DPPA3 (IF)	KLF4 (scRNA-seq); DAZL (qPCR); DDX4 (IF)	BMP15, SYCP3 (qPCR, IF); ZP3 (IF)	hPGCLCs hierarchically cluster separately from PSCs	None	Differentiation to follicle-like structures with morphology of granulosa cells and COCs, and metaphase II oocyte-like cells	Some oocyte-like cells spontaneously developed into cleavage stage-like pre-implantation embryos

a Minor increase in expression and/or depending on cell line.

iPSC: induced pluripotent stem cell; ESC: embryonic stem cell; PSC: pluripotent stem cell; hPGCLC: human primordial germ cell-like cell; hPGC: human primordial gem cell; EB: embryoid bodies; 2D: two dimensional; 3D: three dimensional; qPCR: quantitative polymerase chain reaction; IF: immunofluorescence; (sc)RNA-seq: (single-cell) RNA sequencing; DEGs: differentially expressed genes; GO: gene ontology; 5(h)mC: 5-(hydroxy)methylcytosine; ECM: extracellular matrix; ActA: activin A; WPF: weeks post fertilization.

### Original hPGCLC protocols

We first addressed the details of the 24 original protocols regarding cell type (and cell lines) used as starting material (Table 1, columns A and B). We observed that out of the 24 studies included, 11 exclusively used hESCs as progenitor cells for hPGCLC differentiation and 4 exclusively used hiPSCs. The remaining nine studies included both hESCs and hiPSCs. In addition, the majority of the protocols used multiple cell lines (21 out of 24), with most of these studies using both XY and XX cell lines (13 out of 21). A second variable between protocols concerned the pre-treatment that was applied to the cells, with 13 out of the 24 studies analyzed containing such a step (Table 1, column C). Three of these induced/maintained iPSC naïve pluripotency with use of 4i inhibitor set (inhibitors of Rho-associated protein kinase (iRock), TGFB, MEK, and GSK3) (note: iPSCs correspond to an epiblast-like state and not to a fully naïve state); two protocols derived the hESCs lines in the presence of activin A (ActA); two protocols included a so-called adaptation period of the hESCs/hiPSCs to mouse embryonic fibroblast-conditioned media (MEF-CM); five protocols allowed for the formation of specific structures with the hPSCs, either through the formation of EBs, microwell patterning, adherent culture, or basement membrane extract (BMEx) overlay; one protocol first differentiated hESCs to epiblast-like stem cells (EpiLCs) to achieve primed pluripotency; and one protocol reset hESCs to a naïve pluripotency state.

Another variable considered was the intermediate cell type differentiation or structure formation before final differentiation to hPGCLCs, included in 12 out of the 24 studies (Table 1, columns D–F). Six of these included two-dimensional (2D) (incipient) mesoderm induction; four protocols entailed EB formation in combination with specific growth factor addition; one protocol contained a pre-induction period using 2D culture; one protocol comprised posterior epiblast induction in 2D culture. The duration of the intermediate step of the protocols ranged between 12 h and 72 h, with most studies describing a duration between 24 h and 48 h. The growth or supporting factors added to the media at this stage also differed between protocols, with the most commonly added growth factor being ActA, utilized in nine of the 12 studies. The great majority of these protocols (10 out of 12) also integrated a medium factor to enhance the survival of the progenitor stem cells after dissociation to single-cell suspension (iRock/Y-27632).

The final differentiation step involved EB culture systems in most of the studies (13 out of 24; Table 1, columns G–I) or a mixed system with part EB and part 2D culture system (3 out of 24). One of these protocols continued with maturation in hindgut organoids after EB formation. The remaining protocols utilized a 2D or adherent culture system to proceed with the differentiation. The duration of the final differentiation step ranged from 3 up to 30 days, with most protocols running up to 14 days (19 out of 24). Regarding addition of media components, 16 protocols implemented a static approach (one-step medium) with the culture media, while 8 protocols opted for a dynamic multi-step approach through the addition or removal of growth and induction factors at certain timepoints during the culture. The vast majority of the protocols used BMP4/2 for hPGCLC induction (20 out of 24). In three studies where this was not the case, retinoic acid was used, while one study did not add any additional growth factors to the culture medium. Three other growth factors included in more than half of the studies were LIF, EGF, and SCF (15 out of 24), and these were used as combination of all three in most of these studies (11 out of 15).

Next, we analyzed the differentiation outcomes of the 24 primary studies according to the type of data reported (Table 2). There were 19 studies that reported the use of FACS to assess the percentage of hPGCLCs based on surface markers or reporter gene expression. Of these, six articles opted to use a reporter cell line for sorting, while the others utilized IF for endogenously expressed surface markers. In four articles, a combination of ITGA6 and EpCAM antibodies was used. Other antibodies that were used by the remaining articles included for KIT, ALPL, SSEA1 (or FUT4), and CD38. Furthermore, one article opted to assess differentiation efficiency through immunocytochemistry instead of flow cytometry (Table 2, column A).

In addition, we analyzed the expression of markers to identify hPGCLCs (Table 2, columns B–D). All 24 studies reported expression of known germ cell markers. Of these, 22 articles observed expression and upregulation of markers associated with pluripotency and pre-migratory hPGC stages; 19 articles reported expression and upregulation of markers associated with migratory and post-migratory hPGC stages; 9 articles also showed expression and upregulation of markers associated with meiotic and post-meiotic stages. Although the methodologies varied, all studies measured RNA levels either by microarray, RNA-seq, or qPCR techniques. Furthermore, 21 studies additionally performed IF to assess protein expression for the marker genes.

A more comprehensive analysis of the molecular signature of hPGCLCs can be obtained using RNA-sequencing (RNA-seq) (Table 2, column E). There were 14 articles that provided RNA-seq results, through application of microarray, bulk RNA-seq or single-cell RNA-seq techniques. Ten studies integrated data from both *in vivo* germ cells and *in vitro* differentiated hPGCLCs.

An additional read-out included in 10 studies was information on the epigenetic state of the differentiated hPGCLCs (Table 2, column F). The articles provided data on 5mC and/or on 5hmC modification of the DNA, certain histone modifications, and analyses of expression of methylation-associated genes, obtained through different experimental techniques (e.g. RNA-seq, IF, bisulfite sequencing). Only genes of interest that were specifically shown in main figures or elaborated on in the text were taken into account for this review, hence excluding a deep analysis of the supplementary materials or the raw sequencing data.

Table 2 also includes data on functional analysis and extra remarks in relation to the specific study (Table 2, columns G and H). Fourteen articles (out of 24) provided remarks on functionality related to signaling pathway regulation, gene ontology, particular gene expression patterns, or specific protocol details (efficiency or adaptation period requirement).

### Extension protocols

Extension of a previously published hPGCLC differentiation protocol was described in a total of eight articles (Table 3). The data referring to the extension protocols are summarized using similar parameters as presented in Tables 1 and 2, with citations to the original protocol articles for each study. Seven studies used hiPSCs as starting cell type, with one of those using hESCs as well, while one study used hESCs exclusively (Table 3, column A). Most articles reported the use of multiple cell lines (six out of eight), and one article did not indicate the cell lines included (Table 3, column B).

**Table 3.** Protocol set up and differentiation outcomes of selected extended studies.

Article	Steps	Protocol setup							Differentiation outcomes						
		A. *Cell type*	B. *Cell line(s)*	C. *Extension to*	D. *Description*	E. *Induction / growth factors*	F. *Duration*	G. *FACS (ab/reporter/marker)*	*Markers expression*			K. *Sequencing*	L. *Epigenetic landscape*	M. *Remarks on functionality*	N. *Notes*
									H. *Pluripotency and pre-migratory*	I. *Migratory and post-migratory*	J. *Meiotic and post-meiotic*				
25. Gell *et al.*, 2020	Extended culture	ESC	UCLA2 (XY, ESC), UCLA6 (XY, ESC)	Sasaki *et al.*, 2015 *(removal of SCF, altered duration of intermediate induction)*	[Extended culture] 2D culture on STO feeder cells (GK10 + 50 ng/ml primocin), intermediate hPGCLC purification through FACS	100 ng/ml SCF, 10 μM forskolin, 10 μM rolipram	Up to 21 days	[hPGCLC differentiation] ITGA6+ EpCAM+, no percentages/figures	BLIMP1, TFA2PC, SOX17 (qPCR, IF); NANOS3 (qPCR)	None	None	Initial hPGCLCs and extended culture hPGCLCs clustered together, separately from PSC/intermediate and naïve; identity of extended culture hPGCLCs corresponds to early stage hPGCs (bulk RNA-seq)	TET-genes upregulation (to similar level as hPGCs); H3K27me3, H3K9me2, UHRF1, DNMT3B, DNMT3L downregulation (vs somatic)	GO showed upregulation of genes associated with extracellular matrix and multiple cell signaling pathways	Expansion in STO feeders promotes hPGCLCs proliferation (Ki67 marker analysis)
26. Mall *et al.*, 2020		ESC, iPSC	H9 (XX, ESC), SPF.5/GFP (XY, iPSC), 405.8/GFP (XY, iPSC), CD43/GFP (XY, iPSC)^a^	Sasaki *et al.*, 2015	[Extended culture] Co-culture with rat fetal testicular cells in xeno-organoids (DMEM + 2% KSR)	10 μM iRock	10 days	[Xeno-organoid extension] Up to 30% of human cells (ubiquitously expressing GFP) of which 97% were TNAP+	OCT4, SOX17, TFAP2C (qPCR, IF); NANOS3, BLIMP1, DPPA3 (qPCR)	None	None	None	None	None	Created a model to mimic testicular niche environment
27. Murase *et al.*, 2020		iPSC	585B1 BTAG (XY, iPSC), 1383D6 (XY, iPSC)	Sasaki *et al.*, 2015	[Extended culture] 2D culture on m220–5 inactivated feeder cells (DK15^b^)	10 μM forskolin, 100 ng/ml SCF, 20 ng/ml bFGF	Up to 120 days	[LTC-hPGCLC extension] Up to 40% at D20 and 20% at D120, BLIMP1+(reporter) TFAP2C+(reporter)	BLIMP1, TFAP2C, SOX17, OCT4, NANOG (qPCR, IF, bulk RNA-seq)	None	None	All cell types clustered separately (PSCs, iMeLCS, initial hPGCLCs, LTC-PGCLC) (bulk RNA-seq)	5mC decrease (vs initial hPGCLCs); DNMT3A and DNMT3B downregulation (vs PSCs); No downregulation of UHRF1	GO analysis suggests repression of somatic program that is activated simultaneously as hPGCLC specification (in LTC-PGCLCs)	Karyotypically normal initial hPGCLCs could be propagated nearly 1*10^6 fold in 120 days
28. Kobayashi *et al.*, 2022		iPSC	A4 (XY, iPSC), A5 (XY, iPSC), ASC-1029 (XX, iPSC), 9A13	Mitsunaga *et al.*, 2017	[Extended culture] 2D culture on 1: STO feeder cells (GK13) and 2: no feeder layer (GK13 STO-conditioned media)	100 ng/ml SCF	1: Until 20% confluent and 2: Up to 120 days total	[LTC-hPGCLC extension] Up to 95.1% CD38+ and up to 94.5% TFAP2C+BLIMP1+	BLIMP1, TFAP2C, SOX17 (bulk RNA-seq, IF); OCT4 (IF); DPPA3 (bulk RNA-seq)	PIWIL2 (bulk RNA-seq)	None	LTC-hPGCLCs cluster together with initial hPGCLCs and separately from iPSCs (bulk RNA-seq and scRNA-seq)	H3K27me3 upregulation and H3K9me2 downregulation (vs PSCs); 5mC decrease (vs PSCs/initial hPGCLCs)	scRNA-seq showed homogeneity and active proliferation of LTC-hPGCLCs; use of xrTestis culture model to show maturation potential of LTC-hPGCLCs	None
29. Hwang *et al.*, 2020	Maturation	iPSC	not mentioned	Sasaki *et al.*, 2015 (altered duration of intermediate induction)	[Maturation] Co-culture through xrTestis formation with mouse fetal testicular somatic cells (E12.5) in 1: 96U-well plate (αMEM + 10% KSR) and 2: Transwell (αMEM + 10% KSR)	1: 10 μM iRock and 2: none	1: 2 days and 2: 120 days	[xrTestis extension] TFAP2C+(reporter); no cells positive for DDX4 (reporter)	TFAP2C, OCT4, NANOG, SOX17 (IF, scRNA-seq); BLIMP1, NANOS3 (scRNA-seq)	DDX4, DAZL (IF, scRNA-seq); DPPA3 (scRNA-seq)	None	Cell types clustered separately (PSCs, iMeLCs, initial hPGCLCs) with exception of matured hPGCLCs, which clustered together as sub-clusters (scRNA-seq)	None	GO analysis showed enrichment of genes involved in spermatogenesis, DNA methylation involved in gamete generation and piRNA metabolic processes	Higher expression of genes related to human infertility in matured hPGCLCs
30. Yamashiro *et al.*, 2018		iPSC	585B1 BTAG (XY, iPSC), 1390G3/1390/G3 AGVT (XX, iPSC)	Sasaki *et al.*, 2015	[Maturation] Co-culture through xrOvary formation with mouse fetal ovarian somatic cells (E12.5) in 1: 96U-well plate (GK15^c^) and 2: transwell (αMEM)	1: 10 μM iRock and 2: 150 nM L-ascorbic acid	1: 2 days and 2: 120 days	[hPGCLC differentiation] BLIMP1+(reporter) TFAP2C+(reporter), percentages not shown	OCT4, BLIMP1, TFAP2C, SOX17 (qPCR, IF, bulk RNA-seq); NANOS3, DPPA3 (qPCR, bulk RNA-seq)	DDX4 (qPCR, IF, bulk RNA-seq); DAZL (qPCR, bulk RNA-seq)	SYCP3 (IF)	xrOvaries showed similarity to week 7 and 9 oogonia and gonocytes. Matured hPGCLC cluster separately from initial hPGCLCs, PSCs and iMeLCs (bulk RNA-seq)	5mC decrease, comparable to oogonia and gonocytes at embryo weeks 7–10 (vs PSC)	Upregulation of BMP and retinoic acid responsive genes	Partial reactivation (biallelic expression) of several X-linked genes, but no reactivation of *XIST*
31. Arkoun *et al.*, 2022	Maturation	iPSC	PB12.CO3 (XX, iPSC)	Irie *et al.*, 2015 *(removal of intermediate induction)*	[Maturation] EB in hanging drops (DK15^b^)	100 ng/ml BMP4, 20 ng/ml SCF, 1 μM retinoic acid	6 days	[hPGCLC differentiation] Up to 31.2% PDPN+	PDPN (qPCR, IF); SOX17, CD38, NANOS3 (qPCR); TFAP2C, OCT4 (IF, scRNA-seq); BLIMP1 (scRNA-seq)	DDX4 (qPCR, IF); KIT, DAZL^d^ (qPCR)	SYCP3 (qPCR, IF); MEIOC, DMC1, STRA8, (qPCR)	Three sub-clusters of PGCLCs, defined by different levels of TET1 expression (scRNA-seq of exclusively initial hPGCLCs)	TET1 upregulation (vs somatic)	Early hPGCLC sub-clusters indicated active BMP signaling and downregulation of retinoic acid signaling	The initial hPGCLC state did not progress when co-cultured with ovary material
32. Murase *et al.*, 2024		iPSC	585B1 BTAG (XY, iPSC), 1383D6 (XY, iPSC), 1390G3 (XX, iPSC), NCLCN (XX, iPSC)	Sasaki *et al.*, 2015	[Maturation] 2D culture (RPMI + 15% KSR + 1X GlutaMAX + 1% P/S + 0.1 mM β-mercaptoethanol + 2.5% FBS) on 1: m220–5 inactivated feeder cells for the first passage and 2: no feeder cells	1: 100 ng/ml SCF, 10 μM forskolin, 20 ng/ml bFGF, 1.5 μM IWR1, 25 ng/ml BMP2 (male) or 50 ng/ml BMP2 (female) and 2: 100 ng/ml SCF, 10 μM forskolin, 20 ng/ml bFGF, 1.5 μM IWR1,100 ng/ml BMP2	Up to 142 days	[LTC-hPGCLC extension] Up to 81.9% TFAP2C+ DAZL+ and up to 80.8% TFAP2C+ DDX4+ at D140	OCT4, BLIMP1, TFAP2C, SOX17, NANOS3, NANOG, DPPA3 (scRNA-seq)	DAZL, DDX4, PIWIL2, CDH5, DMRT1 (scRNA-seq)	SYCP3 (scRNA-seq)	LTC-hPGCLCs clustered with *in vivo* mitotic pro-spermatogonia or oogonia and *in vitro* oogonia-like cells in xrOvaries; female hPGCLCs trajectory resembles 7 WPF until 13/16 WPF female germ cells *in vivo* (scRNA-seq)	5mC levels decreased to ∼10%; LTC-hPGCLCs upregulated epigenetic reprogramming-genes; DNMT3B and UHRF1 downregulation (vs iPSCs); replication-coupled passive genome-wide DNA demethylation	GO analysis showed downregulation of cell adhesion and extracellular matrix organization; X chromosome reactivation partially occurs during the culture	Suggest that expression of epigenetic reprogramming genes occurs as response to promoter demethylation

a EF1a-GFP tagged (ubiquitously expressed).

b DK15: DMEM/F12, 15% KSR, 0.1 mM NEAA, 2 mM GlutaMax, 1 mM sodium pyruvate, 0.1 mM β-mercaptoethanol.

c GK15: GMEM, 15% KSR, 1× NEAA, 2 mM L-glutamine, 1 mM sodium pyruvate, 0.1 mM β-mercaptoethanol.

d Minor increase in expression and/or depending on cell line.

iPSC: induced pluripotent stem cell; ESC: embryonic stem cell; PSC: human pluripotent stem cell; hPGCLC: human primordial germ cell-like cell; EB: embryoid bodies; 2D: two dimensional; 3D: three dimensional; LTC-hPGCLC: long-term culture hPGCLC; iMeLCs: incipient mesoderm-like cells; KSR: knockout serum replacement; STO: mouse embryonic fibroblast cell line; BMP: bone morphogenetic protein; bFGF: basic fibroblast growth factor; SCF: stem cell factor; IWR: WNT inhibitor; iRock: Rock inhibitor (Y-27632); P/S: penicillin-streptomycin; DMEM: Dulbecco’s modified eagle medium; GMEM: Glasgow’s eagle minimum essential medium; αMEM: minimum essential medium eagle alpha modification; FBS: fetal bovine serum; xrOvary: xenogeneic reconstituted ovary; xrTestis: xenogeneic reconstituted testis; qPCR: quantitative polymerase chain reaction; IF: immunofluorescence; (sc)RNA-seq: (single-cell) RNA sequencing; 5(h)mC: 5-(hydroxy)methylcytosine; GO: gene ontology; LTC: long-term culture; WPF: weeks post fertilization.

The extended protocols were grouped according to their goals. Four protocols indicated the goal of long-term-culture period for hPGCLCs, with a duration ranging between 10 and 120 days (Table 3, columns D–F). The majority of these studies opted for a 2D culture system (three out of four), while one protocol employed a xeno-organoid technique (Table 3, column D). Regarding the use of growth factors or induction factors, the use of SCF was reported by most of the studies (three out of four; Table 3, column E). The four remaining studies presented in Table 3, described germ cell maturation as their goal, with a duration ranging between 10 and 142 days (Table 3, columns D–F). Three of these reported the use of a 3D culture system and one used a 2D culture system partially using a feeder layer. All protocols used a distinct combination of growth factors (or concentrations), such as BMP4, SCF, ascorbic acid, and retinoic acid (Table 3, columns D and E). Additionally, both long-term-culture and maturation extension protocols included the use of a supporting cell type or feeder layer (seven out of eight). However, most long-term-culture protocols resorted to the use of a feeder layer of STO cells (cell line established from sandos inbred mice embryonic fibroblasts) or m220-5 cells (mouse cell line mutated to express a membrane-bound form of SCF) (three out of four). Only the maturation protocols integrated the use of mouse fetal gonadal somatic cells (two out of four) (Table 3, column D).

Regarding the differentiation outcomes, all long-term-culture protocols reported expression of known markers associated with pluripotency and pre-migratory hPGC stages, with one study indicating expression of a marker associated with migratory and post-migratory hPGCs. As for the studies focused on germ cell maturation, all of them reported expression of markers associated with migratory and post-migratory hPGC stages, while three out of four also reported expression of markers known to be specifically expressed in meiotic and post-meiotic germ cell stages (Table 3, columns H–J). All studies assessed the expression of markers by measuring RNA levels, either through RNA-seq or qPCR, alongside IF to assess protein presence.

Regarding RNA-seq data, seven articles provided sequencing results, through application of bulk RNA-seq or single-cell RNA-seq. Most of the studies demonstrated a distinct clustering of hPGCLCs resulting from the initial protocol compared to the extension protocol. Two studies integrated data from *in vivo* germ cells as a comparison to data from the *in vitro* differentiated germ cells (Table 3, column K).

Additionally, six articles reported data on the epigenetic state of the differentiated hPGCLCs, such as data on 5(h)mC modification of the DNA, certain histone modifications, and expression of methylation-associated genes, obtained through different experimental techniques (e.g. RNA-seq, IF, bisulfite sequencing; Table 3, column L).

Finally, data on functional analysis and extra remarks on signaling pathway regulation, gene ontology, particular gene expression patterns, or specific protocol details (trial with advanced culture models to assess maturation potential) were also provided (Table 3, columns M and N).

## Discussion

In the past decades, several studies have established protocols to differentiate hPGCLCs from hiPSCs and/or hESCs, using different culture media and reporting different efficiencies and functional outcomes. Furthermore, culture parameters also differed during the differentiation of hPGCLCs to migratory or post migratory germ cells. Here we discuss the theoretical context for the main parameters used, and the validity of the assessment on the degree of success. We highlight the similarities and differences between the various protocols and discuss potential associations between protocol setups and differentiation outcomes.

### BMP is the main, but not the only, inducer of hPGCLC differentiation

The initial induction of the germ cell lineage is a critical event for the success of hPGCLC differentiation. For this, the use of the appropriate growth and induction factors plays an essential role. Although many factors were described, the results highlight the importance of BMP4, used in the great majority of the protocols (Table 1). Nine of these studies (Table 1, Protocols 7–9, 11, 13, 14, 20, 21, 23) also opted to include WNT3A or CHIR99021 (a GSK3 inhibitor that prevents degradation of β-catenin), both of which will lead to the activation of the WNT/β-catenin pathway (Peischard *et al.*, 2017). One protocol was able to induce nascent hPGCLCs without use of growth factors, resorting only to the addition of BMEx to the media (Table 1, Protocol 16), challenging the need to use exogenous BMP. Nevertheless, the majority of the reported data aligns with the knowledge that BMP signaling is crucial to activate the germ cell program, interplaying with other pathways such as WNT and NODAL to induce expression of key transcription factors, such as BLIMP1 (Jo *et al.*, 2022; Murase *et al.*, 2024). The results also highlight the use of retinoic acid to induce hPGCLC differentiation in protocols where BMP was not present (Table 1, Protocols 5, 14, 19). Retinoic acid is known to influence spermatogonial differentiation and germ cell meiosis by regulating the expression of STRA8, a gene essential for meiotic initiation (van Pelt and de Rooij, 1991; Zuo *et al.*, 2019). However, its precise role in the initial hPGCLC induction is not fully understood, with evidence suggesting a role in the activation of the WNT pathway and regulation of the epigenome (Cheng *et al.*, 2017; Zuo *et al.*, 2019). Regarding the extension protocols, BMP was only used in two studies (Table 3, Protocols 31, 32). Instead, most extension protocols added forskolin, ascorbic acid, or retinoic acid, which have a direct impact on cAMP activation and gene expression, epigenetic regulation through interaction with TET enzymes, and signaling pathway and gene activation, respectively (Tedesco *et al.*, 2013; Bahmanpour *et al.*, 2020; Li *et al.*, 2019; Murase *et al.*, 2024) (Table 3, Protocols 25, 27, 30). In one article, rolipram was used together with forskolin (Table 3, Protocol 25). Although both factors are cAMP activators, rolipram has also been reported to stimulate mPGCLC proliferation (Ohta *et al.*, 2017; Li *et al.*, 2019). SCF was used in most extended culture protocols (Table 3, Protocols 25, 27, 28, 31, 32). This correlates with the importance of this factor *in vivo*, which was reported to be required for migration, regulation of PGC survival, and proliferation in mice (Gu *et al.*, 2009). Altogether, this could indicate that, while necessary for the initial induction period, BMP signaling is not required for extended maturation or long-term culture of hPGCLCs. Instead, the addition of factors that can act on the epigenetic reprogramming of the germ cells might be preferential after hPGCLC specification.

Another parameter to consider is the concentration of the growth factors. Although BMP4 was predominantly used, the concentration ranged from 10 ng/ml to 500 ng/ml (Table 1). Since BMP4 is a morphogen, different concentrations of BMP4 will impact hPGCLC differentiation and affect its efficiency (Overeem *et al.*, 2023; Table 1, Protocol 17). Furthermore, the timing of exposure of the cells to growth factors is also a critical variable for hPGCLC differentiation, as was shown for BMP and NODAL (Jo *et al.*, 2022; Table 2, Protocol 20). Differences in timing can affect the activity of signaling pathways, such as WNT, where the temporal regulated activation and its sequential inhibition directly affected efficiency and promoted hPGCLC specification (Vijayakumar *et al.*, 2023). For this reason, it is fundamental to adapt the combination, concentration and timing of the growth factors to the specific protocol employed, so that each protocol can be optimized to its maximum efficiency.

### Cell lines used show different differentiation capacity

Many of the articles discussed used multiple cell lines, showing that the differentiation efficiency can vary depending on the cell line used. Some protocols used the same (gene-edited) cell lines, for example, the ‘BTAG’ line, harboring the BLIMP1::tdTomato and TFAP2C::EGFP reporters (Tables 1 and 3, Protocols 9, 27, 30, 32). Although using the same cell lines is a pragmatic approach for facilitating comparisons between protocols, it can raise the question whether the success of the protocol is due to the particular cell lines’ inherent propensity for hPGCLC differentiation. In addition, different clones (with different genome integrities), different culture conditions, and a different passage number at the start of the differentiation procedure could be of importance for the final outcome. For example, cell line editing and prolonged culture can have adverse effects on their potential (Choi *et al.*, 2017; Poetsch *et al.*, 2022). Furthermore, the source of the cells used for reprogramming to generate hiPSCs can impact the differentiation potential of the cells (Yokobayashi *et al.*, 2017; Chang *et al.*, 2021). One article reported that hiPSCs with high expression of a specific group of genes, including NODAL, displayed a higher potential for hPGCLC differentiation compared to cells with lower expression of these genes (Overeem *et al.*, 2023). Another factor to consider is the use of XX or XY cells, as XX cells are known to be more vulnerable in terms of X chromosome-derived gene expression dosage in hPSCs (Mekhoubad *et al.*, 2012), due to the erosion of epigenetic marks on the silent X chromosome with passaging. Working with female cells requires routine testing of X chromosome status, and advances in generating high quality XX hiPSC lines are necessary (Volpato and Webber, 2020). Altogether, to be able to show that the protocol for hPGCLC specification is universal, a variety of cell lines with distinct sex and genetic backgrounds should be used.

### Relevance of pre-treatment and intermediate cell states during differentiation

Most studies included either a pre-treatment step or transition to an intermediate cell state, before proceeding to hPGCLC differentiation. In some cases, particularly when using MEF-CM, the pre-treatment ensured the survival of the hESCs during the transition to a feeder-free culture system, instead of being directly associated with the differentiation process (Villa-Diaz *et al.*, 2009). Another case revealed that a priming period to stimulate structure formation was essential for the hPGCLC differentiation (Overeem *et al.*, 2023). The derivation of hESC lines in the presence of ActA was also reported to improve differentiation competence to hPGCLCs compared to control lines (Table 2, Protocols 2, 15). This aligns with the knowledge that ActA influences the transcriptional signature of hESCs, thereby increasing their competence for differentiation into the germ cell lineage (Mishra *et al.*, 2021). Another methodology adopted was the use of 4i-medium to convert or retain progenitor cells in a naïve pluripotency associated state (Peischard *et al.*, 2017) (Table 1, Protocols 3, 6, 13). This improves the differentiation potential to hPGCLCs, possibly by maintaining these cells in a pluripotency state better correlated to the inner cell mass in blastocysts (Irie *et al.*, 2015; Table 1, Protocol 6). This methodology is particularly efficient if paired with ActA supplementation, leading to further improvement of the hESCs competence to differentiate into hPGCLCs (Mishra *et al.*, 2021).

The most adopted intermediate step identified in our search was the induction of incipient mesoderm-like cells (Table 1, Protocols 7–11, 20). One protocol mentions the induction of posterior epiblast and describes the same conditions as for the mesoderm induction (Table 1, Protocol 21). For discussion purposes, we considered them to be a similar intermediate cell type. This transition depended on the activation of specific signaling pathways, such as BMP, WNT, and NODAL which are also critical for the initial induction of the germ cell transcriptional network *in vitro* (Jo *et al.*, 2022; Murase *et al.*, 2024). This way, the culture system represents what occurs in *in vivo* development and allows for better control over the differentiation process (Vijayakumar *et al.*, 2023). Although the two previously mentioned strategies describe differentiation to distinct cell types (mesoderm-like cells and 4i-induced naïve pluripotent cells), literature has reported that these share, in fact, a transcriptomic profile reminiscent of a peri-gastrulation stage, distinct from naïve pluripotency (Alves-Lopes *et al.*, 2023). A distinct strategy relied on the use of reset hPSCs (rhPSCs), representative of an intermediate pluripotency state between primed and naïve, for hPGCLC differentiation. The differentiated hPGCLCs presented transcriptomic differences dependent of their distinct precursors. This is observed in terms of decrease of 5mC and H3K9me2, and in the lower expression of somatic genes in reset derived-hPGCLC, which could explain their accelerated progression in culture (Alves-Lopes *et al.*, 2023; Table 1, Protocol 1). To identify the optimal precursor for germ cell differentiation, it is fundamental to apply all of them (4i, mesoderm-like, reset) to multiple protocols and verify their universal competence for hPGCLC differentiation.

The duration of the pre-treatment or intermediate steps varies between studies, even within the same type of induction. In the case of intermediate mesoderm induction, different studies indicate distinct treatment windows and state the importance of these to sustain hPGCLC differentiation (Sasaki *et al.*, 2015 (Table 1, Protocol 9, 42–48 h), Kobayashi *et al.*, 2017 (Table 1, Protocol 7, 12 h)). These discrepancies indicate that it may be essential to optimize the timing of each step when a particular protocol is employed while taking into consideration all other culture condition variables (cell lines, growth factor, structure).

Overall, intermediate steps, with addition of growth factors or a priming period, play an important role in the success of hPGCLC differentiation, allowing better fine tuning of the protocols. However, there does not seem to be a clear correlation between the type of pre-treatment or intermediate differentiation adopted and the reported results regarding differentiation efficiency and hPGCLC maturation status.

### 2D versus 3D culture systems: does structural organization matter?

The data collected revealed no clear preference between the protocols on the use of either a 2D (e.g. monolayer, multilayer, and micropattern culture systems) or a 3D culture platform (EBs or cellular aggregates) (Tables 1 and 2). On one occasion, the single use of a BMEx overlay, without the addition of other factors, allowed for the recreation of a multilayer amniogenic environment that led to successful induction of hPGCLCs. This underlines the importance of structural organization, independent of use of exogenous induction factors, due to the influence it exerts in modulating cellular exposure to signaling cues (Esfahani *et al.*, 2024; Table 1, Protocol 16). Either by controlling colony size or employing a multilayer cell-ECM structure, it is possible to increase differentiation efficiency substantially and reduce the concentration of growth factors required for hPGCLC differentiation (e.g. BMP4), in comparison to 3D systems (Jo *et al.*, 2022 (Table 2, Protocol 20); Overeem *et al.*, 2023 (Table 2, Protocol 17)). This data indicate that the 2D protocols offer a simplified and more efficient methodology, in comparison to 3D systems, and can offer a higher potential for scalability of the differentiation. On the other hand, the majority of studies that reported expression of later markers or extension protocols for hPGCLC maturation employed a 3D system during hPGCLC differentiation or subsequent maturation. This could indicate that, in comparison to 2D cultures, 3D systems show an increased potential to promote further maturation of the differentiated hPGCLCs. It should be noted, however, that five of these seven protocols are extensions to the same protocol (Sasaki *et al.*, 2015; Table 1, Protocol 9), using a 3D EB-based approach, explaining the choice for a 3D system for further culture. Consequently, considering the benefits of initial 2D-based hPGCLC differentiation protocols, an optimal approach could consist of the combination of a 2D system for hPGCLC specification, followed by a switch to a 3D system for further maturation. The use of a 3D structure, possibly integrating co-culture with gonadal soma, might allow for a better mimicking of the *in vivo* environment and progression of the germ cells to a later stage.

### Effects of somatic co-culture environment on hPGCLC differentiation

The extension protocols reviewed utilized different types of co-cultures, including an STO-feeder layer, MEFs, and 3D aggregation of primary mouse or human somatic cells with the differentiated hPGCLCs (Table 3). Considering most extension protocols had to implement a type of co-culture to reach a later stage or proliferation, it seems important to recapitulate certain interactions of hPGCLCs with somatic cells *in vitro*. Although in some of these studies, hPGCLCs gained expression of later (peri- and post-migratory) markers, successful completion of meiosis was not yet achieved (Abdyyev *et al.*, 2020). The general inability to reach a complete maturation stage might be due to the pre-migratory state that nascent hPGCLCs are often still in after their *in vitro* specification (Irie *et al.*, 2015; Sugawa *et al.*, 2015; Overeem *et al.*, 2023).

To recapitulate the *in vivo* situation where germ cells mature in the developing gonad, the next step *in vitro* would be the creation of a co-culture system with hPGCLCs and gonadal niche-like cells. Mouse co-cultures of ovarian somatic cells and mPGCLCs as well as of testicular somatic cells and mPGCLCs showed signaling interactions between these cell types, inducing maturation of the germ cell-like cells (Hikabe *et al.*, 2016; Zhou *et al.*, 2016). An earlier developmental stage that could be considered as alternative to the co-culture with gonadal somatic niche is the use of hindgut organoids. By mimicking the embryonic environment through which the hPGCs migrate *in vivo*, this system achieves maturation of hPGCLCs to a migratory state (Alves-Lopes *et al.*, 2023; Table 2, Protocol 1). Still, in a very different setting, the successful differentiation of hPGCLCs into oocytes at MII stage *in vitro* was reported, without resort to co-culture, except for a brief period after purification of the hPGCLCs (Yu *et al.*, 2023; Table 1, Protocol 24). However, the culture system included the use of follicular fluid which, *in vivo*, is produced by granulosa cells and corresponds to the environment in which the germ cell matures (Pan *et al.*, 2024). This suggests that part of the *in vitro* maturation process of germ cells might be possible without resorting to co-culture. However, in this particular culture system both germ and somatic gonadal cells were reported to develop from the same starting cell population and conditions, which is, in that sense, perhaps similar to a co-culture setting.

Combining protocols for obtaining matured post-migratory hPGCLCs (such as those described in Table 3) with co-culturing protocols could aid in achieving full maturation *in vitro*. One solution to overcome the use of primary (mouse or human) material for co-culture would be to differentiate gonadal niche-like cells from hiPSCs *in vitro*, hence creating a full germ cell development culture system from hiPSCs, as recently demonstrated using female mice PSCs (Yoshino *et al.*, 2021).

### *In vitro* versus *in vivo* developmental timelines

*In vivo*, the development of hPGCs is believed to initiate around day 12 post-fertilization, with expression of early markers (e.g. BLIMP1 and SOX17), while the expression of late markers (e.g. SYCP3 and STRA8) is initiated in female germ cells around 10 weeks post-fertilization (Chen *et al.*, 2019; Cheng *et al.*, 2022). In the *in vitro* culture systems, the expression of early and late markers fluctuates between protocols. For example, some studies reported expression of early and late markers as early as days 3 and 6 of differentiation, respectively, while others reported late marker expression only later on, at day 12 of culture (Tables 1 and 2). This could be a reflection of the distinct timepoints setup during hPGCLC development/differentiation or, in fact, of a different developmental stage between the differentiated cells. However, transcriptomic analyses do show evidence of correlation between the *in vitro* and *in vivo* germ cells. Sequencing data from several studies revealed that hPGCLCs cluster together with hPGCs (Table 2, Protocols 1, 3, 6, 9, 16, 17, 20, 21, 23) and matured hPGCLCs showed strong similarities to week 7 and week 9 oogonia and gonocytes (Table 3, Protocols 30, 32). One of these protocols involved more than 17 weeks of maturation of initial hPGCLCs (Table 3, Protocol 30). Shorter protocols (Table 3, Protocol 31) involved only 6 days of maturation, but these cells did not mature further when co-cultured with human ovarian cells. Thus, this indicates that, currently, to recapitulate *in vivo* development and bona fide maturation of hPGCLCs *in vitro*, long periods of culture may be required, similar to the *in vivo* timeline. Moreover, in addition to transcriptomics, other -omics (e.g. metabolome, proteome) should be included in the molecular characterization to allow for a more thorough comparison between *in vivo* and *in vitro* germ cells.

### Importance of DNA methylation in hPGCLCs

The development of hPGCLCs is directly influenced by epigenetic reprogramming events, involving erasure and re-establishment of DNA methylation marks, essential to reset the epigenome to a sex-specific germline state (Gruhn *et al.*, 2023; Vijayakumar *et al.*, 2023). Most of the studies included in this review did not analyze DNA methylation. Some mainly provided the expression of relevant genes for the epigenome regulation, such as UHRF1, DNMTs, and TETs (Tables 2 and 3, Protocols 11, 12, 23, 31). The studies that provided epigenetic findings reported on whole genome DNA methylation, including 5(h)mC levels and differentially methylated regions of genomic imprinted genes, alongside levels of certain histone modifications (Tables 2 and 3, Protocols 1, 3, 4, 6, 8–10, 25, 27, 28, 20, 32). The DNA methylation state has a major role in the differentiation process, as it directly influences the capacity of the cells to silence the somatic genetic program and reinforce the germline identity. Furthermore, it regulates the potential of hPGCLCs to mature into functional germ cells by resetting parental genomic imprinting (Hackett *et al.*, 2018; Murase *et al.*, 2024). A clear example of the importance of the epigenetic influence on the potential for maturation is, in the case of oogenesis, reactivation of the silenced X chromosome to enable correct progression of meiosis (Chuva de Sousa Lopes *et al.*, 2008; Sangrithi *et al.*, 2017). One of the extension protocols described the X chromosome status and reported that, although the progenitor hiPSCs were eroded, later stage hPGCLCs also exhibited X chromosome erosion, characterized by partial biallelic expression and DNA demethylation of the X chromosomes (Yamashiro *et al.*, 2018; Table 3, Protocol 30; also published as separate protocol in Yamashiro *et al*., 2020). For a more complete assessment of germline identity, it is essential to include extensive epigenome analysis, including characterization of the X chromosome state (e.g. X-linked (allele-specific) gene activity and histone modifications), and to compare them to *in vivo* germ cells.

### Characteristics of hPGCLCs

Adequate differentiation into hPGCLCs could be shown by assessing functionality, for example, through a migration assay, a retinoic acid response assay, or the ability to develop further upon co-culture. Aggregation with the gonadal somatic niche is a suitable approach for assessment of functionality, as maturation through co-culture proves the ability of hPGCLCs to respond to signaling from the gonadal somatic niche and to develop further into more mature germ cells. Multiple (extension) protocols implemented xenogeneic reconstituted gonads, co-culturing hPGCLCs with murine gonadal cells, showing that hPGCLCs could mature to a later stage upon long-term culturing (Table 3, Protocols 26, 29, 30). In co-culture with human ovarian cells, one article reported maturation of hPGCLCs (Table 2, Protocol 17), while another reported that the hPGCLCs remained arrested at the early stages (Arkoun *et al.*, 2022; Table 3, Protocol 31). The developmental stage of hPGCLCs, their epigenetic status prior to co-culture, and the developmental stage of the gonadal cells used for co-culture could be critical for their ability to mature further.

In a more indirect type of functional assessment, some articles used transcriptomic analyses in combination with gene ontology (GO) analysis to show enrichment of gene expression, associated with migration and spermatogenesis (Tables 2 and 3, Protocols 3, 6, 9, 16, 25, 27, 29, 32). Some protocols highlighted the activation of certain pathways during hPGCLC differentiation, such as WNT, BMP, activin, and TGFβ (Tables 2 and 3, Protocols 14–16, 20, 25, 30, 31). All in all, it seems advisable to consider functional analyses, since this provides a more complete assessment of cell states, and thus quality assurance of the differentiation state of hPGCLCs.

### Technical differences between protocols affect comparisons between studies

While comparing protocols, a few technical differences became apparent, mainly regarding the analysis of RNA sequencing data. In scRNA-seq, the first steps of the analyses are often subjective because of manual annotation based on known marker gene expression, although automated annotation methods are used more often (Pasquini *et al.*, 2021). After analyses of datasets, many papers compared their data to published *in vivo* datasets or other *in vitro* data, and this includes an extra merging or integration step, to overcome batch differences (Hao *et al.*, 2024). The decision to merge versus integrate impacts the accuracy of the comparisons, as integration is more complex but corrects for batch effects, while merging does not. For the comparisons between scRNA-seq and bulk RNA-seq datasets, often pseudobulk analyses are used. These are generally less heavy computationally and correct for false positives due to within-sample correlations, for example, gene expression patterns that are shared between samples within the same individual. However, their ability to detect differentially expressed genes (DEGs) might be lower compared to full scRNA-seq analyses (Zimmerman *et al.*, 2021; Gagnon *et al.*, 2022; Murphy and Skene, 2022; Heumos *et al.*, 2023). Furthermore, bulk RNA-seq data have the disadvantage that they often represent data from a heterogeneous cell population, making the comparison of one group with another less reliable. In such comparisons, the similarity detected between datasets is directly related to a comparable cell type composition and their relative contributions to the bulk data. Regardless of the type of sequencing, it is important to notice that such analyses reflect gene expression; therefore, validation experiments should confirm the protein expression, when adequate.

Regarding the analysis of differentiation efficiency, the choice of cell surface proteins or reporter genes used for FACS affects the outcome. While some of the antibodies for cell surface proteins recognize early hPGCLCs markers, others (e.g. KIT) recognize epitopes that are representative for a later (migratory) stage of development (Gkountela *et al.*, 2013). Thus, the calculated efficiency could be lower in the latter case, since the collected cell fraction would not include early stage hPGCLCs. The same holds for the reporter genes used: BLIMP1 and TFAP2C are expressed earlier by germ cells than DDX4 (Yamashiro *et al.*, 2018). Hence, when comparing differentiation outcomes between studies, one should take into consideration the cell surface protein or reporter gene used for FACS, and if those include the whole or just a sub-population of hPGCLCs. Moreover, other small technical differences in the application and interpretation of certain procedures, such as differences in IF imaging, are relevant to consider. The presence of certain proteins in the cell is reported in our results (Table 2), but we did not discuss the quality of the IF (antibody specificity and quality control verification), and variability between papers in observed staining patterns. Thus, differences in antibody usage and interpretation of the IF may explain part of the variation in reported results.

### Limitations of the scoping review process

One of the general limitations of this scoping review is the selection of literature: not all papers clearly state whether they are using a newly developed protocol or a previously published protocol. In case an earlier published protocol is used, the reference to the earlier protocol is occasionally missing. Another general limitation is the process of data comparisons. The aim of a scoping review is to systematically compare literature without integrating data and performing re-analyses. Hence, only the conclusions the authors themselves drew in their articles have been included in our analyses. Although these were all peer reviewed papers, the analyses in this scoping review might be susceptible to technical differences between protocols and the interpretations of the authors in their respective studies.

### Future directions

This scoping review provides an elaborate overview comparing protocols to generate hPGCLCs *in vitro*. These comparisons can help the field to make an informed decision on which culture protocol conditions to adopt, depending on the experimental goal. Based on the data collection in this article, we conclude that both 2D and 3D protocols perform well in generating bona fide hPGCLCs. However, for further maturation of these cells, 3D cultures seem to perform better. Hence, a sequential strategy including both systems could be optimal, combining the higher potential for scalability of the 2D systems during the initial stage of differentiation, with an increased maturation potential using the 3D models, supported by gonadal somatic cells. Additionally, the transition through an intermediate cell stage or priming period might offer an opportunity to better control the differentiation to hPGCLCs. The effect of the growth factors used in the protocols with intermediate steps also shifts. While during early differentiation, BMP4 is the most commonly used factor, mimicking *in vivo* specification events, for subsequent maturation steps, there may be a preference for factors associated with epigenetic remodeling. In this regard, it will be important for future studies to provide a more comprehensive molecular signature, including methylome and metabolome, as part of germ cell characterization. In conclusion, further protocol optimization for hPGCLC maturation is still required and will depend on the combination of pretreatment, intermediate, differentiation, and maturation steps, along with more extensive functional assessment and integration of omics analyses. Novel insights into hPGCLC induction and maturation to ultimately establish functional IVG protocols will help us to understand causes of human infertility and may one day play a direct role in human reproduction (de Bruin, 2025).

## Data Availability

No new data were generated or analyzed in support of this research.

## References

[hoaf056-B1] Abdyyev VK , Sant DW, Kiseleva EV, Spangenberg VE, Kolomiets OL, Andrade NS, Dashinimaev EB, Vorotelyak EA, Vasiliev AV. In vitro derived female hPGCLCs are unable to complete meiosis in embryoid bodies. Exp Cell Res 2020;397:112358. 33160998 10.1016/j.yexcr.2020.112358

[hoaf056-B2] Alves-Lopes JP , Wong FCK, Tang WWC, Gruhn WH, Ramakrishna NB, Jowett GM, Jahnukainen K, Surani MA. Specification of human germ cell fate with enhanced progression capability supported by hindgut organoids. Cell Rep 2023;42:111907. 36640324 10.1016/j.celrep.2022.111907 PMC7618081

[hoaf056-B3] Anderson RA , Fulton N, Cowan G, Coutts S, Saunders PT. Conserved and divergent patterns of expression of DAZL, VASA and OCT4 in the germ cells of the human fetal ovary and testis. BMC Dev Biol 2007;7:136. 18088417 10.1186/1471-213X-7-136 PMC2211489

[hoaf056-B4] Arkoun B , Moison P, Guerquin MJ, Messiaen S, Moison D, Tourpin S, Monville C, Livera G. Sorting and manipulation of human PGC-LC using PDPN and hanging drop cultures. Cells 2022;11:3832. 36497094 10.3390/cells11233832 PMC9736549

[hoaf056-B5] Bahmanpour S , Keshavarz A, Zarei Fard N. Effect of different concentrations of forskolin along with mature granulosa cell co-culturing on mouse embryonic stem cell differentiation into germ-like cells. Iran Biomed J 2020;24:30–38. 31454861 10.29252/ibj.24.1.30 PMC6900478

[hoaf056-B8] Canovas S , Campos R, Aguilar E, Cibelli JB. Progress towards human primordial germ cell specification in vitro. Mol Hum Reprod 2017;23:4–15. 27798275 10.1093/molehr/gaw069

[hoaf056-B9] Castillo-Venzor A , Penfold CA, Morgan MD, Tang WW, Kobayashi T, Wong FC, Bergmann S, Slatery E, Boroviak TE, Marioni JC et al Origin and segregation of the human germline. Life Sci Alliance 2023;6:e202201706. 37217306 10.26508/lsa.202201706 PMC10203729

[hoaf056-B11] Chang YW , Overeem AW, Roelse CM, Fan X, Freund C, Chuva de Sousa Lopes SM. Tissue of origin, but not XCI state, influences germ cell differentiation from human pluripotent stem cells. Cells 2021;10:2400. 34572048 10.3390/cells10092400 PMC8466594

[hoaf056-B13] Chen D , Sun N, Hou L, Kim R, Faith J, Aslanyan M, Tao Y, Zheng Y, Fu J, Liu W et al Human primordial germ cells are specified from lineage-primed progenitors. Cell Rep 2019;29:4568–4582.e5. 31875561 10.1016/j.celrep.2019.11.083 PMC6939677

[hoaf056-B14] Cheng H , Shang D, Zhou R. Germline stem cells in human. Signal Transduct Target Ther 2022;7:345. 36184610 10.1038/s41392-022-01197-3 PMC9527259

[hoaf056-B15] Cheng T , Zhai K, Chang Y, Yao G, He J, Wang F, Kong H, Xin H, Wang H, Jin M et al CHIR99021 combined with retinoic acid promotes the differentiation of primordial germ cells from human embryonic stem cells. Oncotarget 2017;8:7814–7826. 27999196 10.18632/oncotarget.13958 PMC5352363

[hoaf056-B16] Choi J , Huebner AJ, Clement K, Walsh RM, Savol A, Lin K, Gu H, Di Stefano B, Brumbaugh J, Kim SY et al Prolonged Mek1/2 suppression impairs the developmental potential of embryonic stem cells. Nature 2017;548:219–223. 28746311 10.1038/nature23274 PMC5905676

[hoaf056-B17] Chuva de Sousa Lopes SM , Hayashi K, Shovlin TC, Mifsud W, Surani MA, McLaren A. X chromosome activity in mouse XX primordial germ cells. PLoS Genet 2008;4:e30. 18266475 10.1371/journal.pgen.0040030 PMC2233679

[hoaf056-B18] Clark AT , Bodnar MS, Fox M, Rodriquez RT, Abeyta MJ, Firpo MT, Pera RA. Spontaneous differentiation of germ cells from human embryonic stem cells in vitro. Hum Mol Genet 2004;13:727–739. 14962983 10.1093/hmg/ddh088

[hoaf056-B19] De Bruin IJ , Spaander MM, Harmsen S, Edelenbosch R, Ploem MC, Dartée N, Cardoso Vaz Santos M, Lakshmipathi M, Mulder CL, Van Pelt AMM et al Stem cell-derived gametes: what to expect when expecting their clinical introduction. Hum Reprod 2025;40:1605–1615. 40605085 10.1093/humrep/deaf123 PMC12408906

[hoaf056-B20] Duggal G , Heindryckx B, Warrier S, O'Leary T, Van der Jeught M, Lierman S, Vossaert L, Deroo T, Deforce D, Chuva de Sousa Lopes SM et al Influence of activin A supplementation during human embryonic stem cell derivation on germ cell differentiation potential. Stem Cells Dev 2013;22:3141–3155. 23829223 10.1089/scd.2013.0024 PMC3856713

[hoaf056-B21] Duggal G , Heindryckx B, Warrier S, Taelman J, Van der Jeught M, Deforce D, Chuva de Sousa Lopes S, De Sutter P. Exogenous supplementation of activin A enhances germ cell differentiation of human embryonic stem cells. Mol Hum Reprod 2015;21:410–423. 25634576 10.1093/molehr/gav004

[hoaf056-B22] Esfahani SN , Zheng Y, Arabpour A, Irizarry AMR, Kobayashi N, Xue X, Shao Y, Zhao C, Agranonik NL, Sparrow M et al Derivation of human primordial germ cell-like cells in an embryonic-like culture. Nat Commun 2024;15:167. 38167821 10.1038/s41467-023-43871-2 PMC10762101

[hoaf056-B23] Gagnon J , Pi L, Ryals M, Wan Q, Hu W, Ouyang Z, Zhang B, Li K. Recommendations of scRNA-seq differential gene expression analysis based on comprehensive benchmarking. Life (Basel) 2022;12:850. 35743881 10.3390/life12060850 PMC9225332

[hoaf056-B24] Gell JJ , Liu W, Sosa E, Chialastri A, Hancock G, Tao Y, Wamaitha SE, Bower G, Dey SS, Clark AT. An extended culture system that supports human primordial germ cell-like cell survival and initiation of DNA methylation erasure. Stem Cell Reports 2020;14:433–446. 32059791 10.1016/j.stemcr.2020.01.009 PMC7066331

[hoaf056-B25] Gkountela S , Li Z, Vincent JJ, Zhang KX, Chen A, Pellegrini M, Clark AT. The ontogeny of cKIT+ human primordial germ cells proves to be a resource for human germ line reprogramming, imprint erasure and in vitro differentiation. Nat Cell Biol 2013;15:113–122. 23242216 10.1038/ncb2638 PMC3786872

[hoaf056-B26] Gomes Fernandes M , Bialecka M, Salvatori DCF, Chuva de Sousa Lopes SM. Characterization of migratory primordial germ cells in the aorta-gonad-mesonephros of a 4.5-week-old human embryo: a toolbox to evaluate in vitro early gametogenesis. Mol Hum Reprod 2018;24:233–243. 29528446 10.1093/molehr/gay011 PMC6018722

[hoaf056-B27] Gruhn WH , Tang WWC, Dietmann S, Alves-Lopes JP, Penfold CA, Wong FCK, Ramakrishna NB, Surani MA. Epigenetic resetting in the human germ line entails histone modification remodeling. Sci Adv 2023;9:eade1257. 36652508 10.1126/sciadv.ade1257 PMC9848478

[hoaf056-B28] Gu Y , Runyan C, Shoemaker A, Surani A, Wylie C. Steel factor controls primordial germ cell survival and motility from the time of their specification in the allantois, and provides a continuous niche throughout their migration. Development 2009;136:1295–1303. 19279135 10.1242/dev.030619

[hoaf056-B29] Guo F , Yan L, Guo H, Li L, Hu B, Zhao Y, Yong J, Hu Y, Wang X, Wei Y et al The transcriptome and DNA methylome landscapes of human primordial germ cells. Cell 2015;161:1437–1452. 26046443 10.1016/j.cell.2015.05.015

[hoaf056-B30] Hackett JA , Huang Y, Gunesdogan U, Gretarsson KA, Kobayashi T, Surani MA. Tracing the transitions from pluripotency to germ cell fate with CRISPR screening. Nat Commun 2018;9:4292. 30327475 10.1038/s41467-018-06230-0 PMC6191455

[hoaf056-B31] Hancock GV , Wamaitha SE, Peretz L, Clark AT. Mammalian primordial germ cell specification. Development 2021;148:dev189217. 33722957 10.1242/dev.189217 PMC7990907

[hoaf056-B32] Hao Y , Stuart T, Kowalski MH, Choudhary S, Hoffman P, Hartman A, Srivastava A, Molla G, Madad S, Fernandez-Granda C et al Dictionary learning for integrative, multimodal and scalable single-cell analysis. Nat Biotechnol 2024;42:293–304. 37231261 10.1038/s41587-023-01767-y PMC10928517

[hoaf056-B33] Hayashi K , Ohta H, Kurimoto K, Aramaki S, Saitou M. Reconstitution of the mouse germ cell specification pathway in culture by pluripotent stem cells. Cell 2011;146:519–532. 21820164 10.1016/j.cell.2011.06.052

[hoaf056-B34] Heeren AM , He N, de Souza AF, Goercharn-Ramlal A, van Iperen L, Roost MS, Gomes Fernandes MM, van der Westerlaken LA, Chuva de Sousa Lopes SM. On the development of extragonadal and gonadal human germ cells. Biol Open 2016;5:185–194. 26834021 10.1242/bio.013847 PMC4823981

[hoaf056-B35] Heumos L , Schaar AC, Lance C, Litinetskaya A, Drost F, Zappia L, Lucken MD, Strobl DC, Henao J, Curion F et al; Single-cell Best Practices Consortium. Best practices for single-cell analysis across modalities. Nat Rev Genet 2023;24:550–572. 37002403 10.1038/s41576-023-00586-w PMC10066026

[hoaf056-B36] Hikabe O , Hamazaki N, Nagamatsu G, Obata Y, Hirao Y, Hamada N, Shimamoto S, Imamura T, Nakashima K, Saitou M et al Reconstitution in vitro of the entire cycle of the mouse female germ line. Nature 2016;539:299–303. 27750280 10.1038/nature20104

[hoaf056-B37] Hwang YS , Suzuki S, Seita Y, Ito J, Sakata Y, Aso H, Sato K, Hermann BP, Sasaki K. Reconstitution of prospermatogonial specification in vitro from human induced pluripotent stem cells. Nat Commun 2020;11:5656. 33168808 10.1038/s41467-020-19350-3 PMC7653920

[hoaf056-B38] Irie N , Lee SM, Lorenzi V, Xu H, Chen J, Inoue M, Kobayashi T, Sancho-Serra C, Drousioti E, Dietmann S et al DMRT1 regulates human germline commitment. Nat Cell Biol 2023;25:1439–1452. 37709822 10.1038/s41556-023-01224-7 PMC10567552

[hoaf056-B39] Irie N , Weinberger L, Tang WW, Kobayashi T, Viukov S, Manor YS, Dietmann S, Hanna JH, Surani MA. SOX17 is a critical specifier of human primordial germ cell fate. Cell 2015;160:253–268. 25543152 10.1016/j.cell.2014.12.013 PMC4310934

[hoaf056-B40] Jo K , Teague S, Chen B, Khan HA, Freeburne E, Li H, Li B, Ran R, Spence JR, Heemskerk I. Efficient differentiation of human primordial germ cells through geometric control reveals a key role for Nodal signaling. Elife 2022;11:e72811. 35394424 10.7554/eLife.72811 PMC9106331

[hoaf056-B41] Kiani M , Movahedin M, Halvaei I, Soleimani M. In vitro differentiation of primed human induced pluripotent stem cells into primordial germ cell-like cells. Mol Biol Rep 2023;50:1971–1979. 36534237 10.1007/s11033-022-08012-w

[hoaf056-B42] Kobayashi M , Kobayashi M, Odajima J, Shioda K, Hwang YS, Sasaki K, Chatterjee P, Kramme C, Kohman RE, Church GM et al Expanding homogeneous culture of human primordial germ cell-like cells maintaining germline features without serum or feeder layers. Stem Cell Reports 2022;17:507–521. 35148847 10.1016/j.stemcr.2022.01.012 PMC9039862

[hoaf056-B43] Kobayashi T , Zhang H, Tang WWC, Irie N, Withey S, Klisch D, Sybirna A, Dietmann S, Contreras DA, Webb R et al Principles of early human development and germ cell program from conserved model systems. Nature 2017;546:416–420. 28607482 10.1038/nature22812 PMC5473469

[hoaf056-B44] Kojima Y , Yamashiro C, Murase Y, Yabuta Y, Okamoto I, Iwatani C, Tsuchiya H, Nakaya M, Tsukiyama T, Nakamura T et al GATA transcription factors, SOX17 and TFAP2C, drive the human germ-cell specification program. Life Sci Alliance 2021;4:e202000974. 33608411 10.26508/lsa.202000974 PMC7918644

[hoaf056-B45] Li L , Dong J, Yan L, Yong J, Liu X, Hu Y, Fan X, Wu X, Guo H, Wang X et al Single-Cell RNA-Seq analysis maps development of human germline cells and gonadal niche interactions. Cell Stem Cell 2017;20:858–873.e4. 28457750 10.1016/j.stem.2017.03.007

[hoaf056-B46] Li Z , Fang F, Zhao Q, Li H, Xiong C. Supplementation of vitamin C promotes early germ cell specification from human embryonic stem cells. Stem Cell Res Ther 2019;10:324. 31730021 10.1186/s13287-019-1427-2 PMC6858754

[hoaf056-B47] Lin IY , Chiu FL, Yeang CH, Chen HF, Chuang CY, Yang SY, Hou PS, Sintupisut N, Ho HN, Kuo HC et al Suppression of the SOX2 neural effector gene by PRDM1 promotes human germ cell fate in embryonic stem cells. Stem Cell Reports 2014;2:189–204. 24527393 10.1016/j.stemcr.2013.12.009 PMC3923219

[hoaf056-B48] Mall EM , Rotte N, Yoon J, Sandhowe-Klaverkamp R, Ropke A, Wistuba J, Hubner K, Scholer HR, Schlatt S. A novel xeno-organoid approach: exploring the crosstalk between human iPSC-derived PGC-like and rat testicular cells. Mol Hum Reprod 2020;26:879–893. 33049038 10.1093/molehr/gaaa067

[hoaf056-B49] Mamsen LS , Brochner CB, Byskov AG, Mollgard K. The migration and loss of human primordial germ stem cells from the hind gut epithelium towards the gonadal ridge. Int J Dev Biol 2012;56:771–778. 23417399 10.1387/ijdb.120202lm

[hoaf056-B50] Mekhoubad S , Bock C, de Boer AS, Kiskinis E, Meissner A, Eggan K. Erosion of dosage compensation impacts human iPSC disease modeling. Cell Stem Cell 2012;10:595–609. 22560080 10.1016/j.stem.2012.02.014 PMC3603710

[hoaf056-B51] Mishra S , Taelman J, Popovic M, Tilleman L, Duthoo E, van der Jeught M, Deforce D, van Nieuwerburgh F, Menten B, de Sutter P et al Activin A-derived human embryonic stem cells show increased competence to differentiate into primordial germ cell-like cells. Stem Cells 2021;39:551–563. 33470497 10.1002/stem.3335 PMC8248136

[hoaf056-B52] Mitsunaga S , Odajima J, Yawata S, Shioda K, Owa C, Isselbacher KJ, Hanna JH, Shioda T. Relevance of iPSC-derived human PGC-like cells at the surface of embryoid bodies to prechemotaxis migrating PGCs. Proc Natl Acad Sci U S A 2017;114:E9913–E9922. 29087313 10.1073/pnas.1707779114 PMC5699045

[hoaf056-B53] Mollgard K , Jespersen A, Lutterodt MC, Yding Andersen C, Hoyer PE, Byskov AG. Human primordial germ cells migrate along nerve fibers and Schwann cells from the dorsal hind gut mesentery to the gonadal ridge. Mol Hum Reprod 2010;16:621–631. 20566702 10.1093/molehr/gaq052

[hoaf056-B54] Murase Y , Yabuta Y, Ohta H, Yamashiro C, Nakamura T, Yamamoto T, Saitou M. Long-term expansion with germline potential of human primordial germ cell-like cells in vitro. Embo J 2020;39:e104929. 32954504 10.15252/embj.2020104929 PMC7604613

[hoaf056-B55] Murase Y , Yokogawa R, Yabuta Y, Nagano M, Katou Y, Mizuyama M, Kitamura A, Puangsricharoen P, Yamashiro C, Hu B et al In vitro reconstitution of epigenetic reprogramming in the human germ line. Nature 2024;631:170–178. 38768632 10.1038/s41586-024-07526-6 PMC11222161

[hoaf056-B56] Murphy AE , Skene NG. A balanced measure shows superior performance of pseudobulk methods in single-cell RNA-sequencing analysis. Nat Commun 2022;13:7851. 36550119 10.1038/s41467-022-35519-4 PMC9780232

[hoaf056-B57] Nikolic A , Volarevic V, Armstrong L, Lako M, Stojkovic M. Primordial germ cells: current knowledge and perspectives. Stem Cells Int 2016;2016:1741072. 26635880 10.1155/2016/1741072 PMC4655300

[hoaf056-B58] Ohta H , Kurimoto K, Okamoto I, Nakamura T, Yabuta Y, Miyauchi H, Yamamoto T, Okuno Y, Hagiwara M, Shirane K et al In vitro expansion of mouse primordial germ cell-like cells recapitulates an epigenetic blank slate. EMBO J 2017;36:1888–1907. 28559416 10.15252/embj.201695862 PMC5494472

[hoaf056-B59] Overeem AW , Chang YW, Moustakas I, Roelse CM, Hillenius S, Helm TV, Schrier VFV, Goncalves M, Mei H, Freund C et al Efficient and scalable generation of primordial germ cells in 2D culture using basement membrane extract overlay. Cell Rep Methods 2023;3:100488. 37426764 10.1016/j.crmeth.2023.100488 PMC10326346

[hoaf056-B60] Pan Y , Pan C, Zhang C. Unraveling the complexity of follicular fluid: insights into its composition, function, and clinical implications. J Ovarian Res 2024;17:237. 39593094 10.1186/s13048-024-01551-9 PMC11590415

[hoaf056-B61] Panula S , Medrano JV, Kee K, Bergstrom R, Nguyen HN, Byers B, Wilson KD, Wu JC, Simon C, Hovatta O et al Human germ cell differentiation from fetal- and adult-derived induced pluripotent stem cells. Hum Mol Genet 2011;20:752–762. 21131292 10.1093/hmg/ddq520 PMC3024045

[hoaf056-B62] Pasquini G , Rojo Arias JE, Schafer P, Busskamp V. Automated methods for cell type annotation on scRNA-seq data. Comput Struct Biotechnol J 2021;19:961–969. 33613863 10.1016/j.csbj.2021.01.015 PMC7873570

[hoaf056-B63] Peischard S , Piccini I, Strutz-Seebohm N, Greber B, Seebohm G. From iPSC towards cardiac tissue-a road under construction. Pflugers Arch 2017;469:1233–1243. 28573409 10.1007/s00424-017-2003-1 PMC5590027

[hoaf056-B64] Poetsch MS , Strano A, Guan K. Human induced pluripotent stem cells: from cell origin, genomic stability, and epigenetic memory to translational medicine. Stem Cells 2022;40:546–555. 35291013 10.1093/stmcls/sxac020 PMC9216482

[hoaf056-B65] Popovic M , Bialecka M, Gomes Fernandes M, Taelman J, Van Der Jeught M, De Sutter P, Heindryckx B, Chuva De Sousa Lopes SM. Human blastocyst outgrowths recapitulate primordial germ cell specification events. Mol Hum Reprod 2019;25:519–526. 31211841 10.1093/molehr/gaz035 PMC6802404

[hoaf056-B66] Roelen BAJ , Chuva de Sousa Lopes SM. Stay on the road: from germ cell specification to gonadal colonization in mammals. Philos Trans R Soc Lond B Biol Sci 2022;377:20210259. 36252219 10.1098/rstb.2021.0259 PMC9574628

[hoaf056-B68] Sangrithi MN , Royo H, Mahadevaiah SK, Ojarikre O, Bhaw L, Sesay A, Peters AH, Stadler M, Turner JM. Non-canonical and sexually dimorphic X dosage compensation states in the mouse and human germline. Dev Cell 2017;40:289–301.e3. 28132849 10.1016/j.devcel.2016.12.023 PMC5300051

[hoaf056-B69] Sasaki K , Nakamura T, Okamoto I, Yabuta Y, Iwatani C, Tsuchiya H, Seita Y, Nakamura S, Shiraki N, Takakuwa T et al The germ cell fate of cynomolgus monkeys is specified in the nascent amnion. Dev Cell 2016;39:169–185. 27720607 10.1016/j.devcel.2016.09.007

[hoaf056-B70] Sasaki K , Yokobayashi S, Nakamura T, Okamoto I, Yabuta Y, Kurimoto K, Ohta H, Moritoki Y, Iwatani C, Tsuchiya H et al Robust in vitro induction of human germ cell fate from pluripotent stem cells. Cell Stem Cell 2015;17:178–194. 26189426 10.1016/j.stem.2015.06.014

[hoaf056-B72] Shono M , Kishimoto K, Hikabe O, Hayashi M, Semi K, Takashima Y, Sasaki E, Kato K, Hayashi K. Induction of primordial germ cell-like cells from common marmoset embryonic stem cells by inhibition of WNT and retinoic acid signaling. Sci Rep 2023;13:3186. 36823310 10.1038/s41598-023-29850-z PMC9950483

[hoaf056-B73] Sugawa F , Araúzo-Bravo MJ, Yoon J, Kim K-P, Aramaki S, Wu G, Stehling M, Psathaki OE, Hübner K, Schöler HR. Human primordial germ cell commitment in vitro associates with a unique PRDM14 expression profile. EMBO J 2015;34:1009–1024. 25750208 10.15252/embj.201488049 PMC4406649

[hoaf056-B74] Tang WW , Dietmann S, Irie N, Leitch HG, Floros VI, Bradshaw CR, Hackett JA, Chinnery PF, Surani MA. A unique gene regulatory network resets the human germline epigenome for development. Cell 2015;161:1453–1467. 26046444 10.1016/j.cell.2015.04.053 PMC4459712

[hoaf056-B75] Tedesco M , Desimio MG, Klinger FG, De Felici M, Farini D. Minimal concentrations of retinoic acid induce stimulation by retinoic acid 8 and promote entry into meiosis in isolated pregonadal and gonadal mouse primordial germ cells. Biol Reprod 2013;88:145. 23636811 10.1095/biolreprod.112.106526

[hoaf056-B76] van Pelt AM , de Rooij DG. Retinoic acid is able to reinitiate spermatogenesis in vitamin A-deficient rats and high replicate doses support the full development of spermatogenic cells. Endocrinology 1991;128:697–704. 1989855 10.1210/endo-128-2-697

[hoaf056-B79] Vijayakumar S , Sala R, Kang G, Chen A, Pablo MA, Adebayo AI, Cipriano A, Fowler JL, Gomes DL, Ang LT et al Monolayer platform to generate and purify primordial germ-like cells in vitro provides insights into human germline specification. Nat Commun 2023;14:5690. 37709760 10.1038/s41467-023-41302-w PMC10502105

[hoaf056-B80] Villa-Diaz LG , Pacut C, Slawny NA, Ding J, O'Shea KS, Smith GD. Analysis of the factors that limit the ability of feeder cells to maintain the undifferentiated state of human embryonic stem cells. Stem Cells Dev 2009;18:641–651. 18764735 10.1089/scd.2008.0010 PMC3133563

[hoaf056-B82] Volpato V , Webber C. Addressing variability in iPSC-derived models of human disease: guidelines to promote reproducibility. Dis Model Mech 2020;13:dmm042317. 31953356 10.1242/dmm.042317 PMC6994963

[hoaf056-B83] von Meyenn F , Berrens RV, Andrews S, Santos F, Collier AJ, Krueger F, Osorno R, Dean W, Rugg-Gunn PJ, Reik W. Comparative principles of DNA methylation reprogramming during human and mouse in vitro primordial germ cell specification. Dev Cell 2016;39:104–115. 27728778 10.1016/j.devcel.2016.09.015 PMC5064768

[hoaf056-B84] Wang X , Liao T, Wan C, Yang X, Zhao J, Fu R, Yao Z, Huang Y, Shi Y, Chang G et al Efficient generation of human primordial germ cell-like cells from pluripotent stem cells in a methylcellulose-based 3D system at large scale. PeerJ 2019;6:e6143. 30643676 10.7717/peerj.6143 PMC6330037

[hoaf056-B85] Wongtrakoongate P , Jones M, Gokhale PJ, Andrews PW. STELLA facilitates differentiation of germ cell and endodermal lineages of human embryonic stem cells. PLoS One 2013;8:e56893. 23457636 10.1371/journal.pone.0056893 PMC3573007

[hoaf056-B86] Xuemei L , Jing Y, Bei X, Juan H, Xinling R, Qun L, Guijin Z. Retinoic acid improve germ cell differentiation from human embryonic stem cells. Iran J Reprod Med 2013;11:905–912. 24639715 PMC3941395

[hoaf056-B87] Yamashiro C , Sasaki K, Yabuta Y, Kojima Y, Nakamura T, Okamoto I, Yokobayashi S, Murase Y, Ishikura Y, Shirane K et al Generation of human oogonia from induced pluripotent stem cells in vitro. Science 2018;362:356–360. 30237246 10.1126/science.aat1674

[hoaf056-B88] Yamashiro C , Sasaki K, Yokobayashi S, Kojima Y, Saitou M. Generation of human oogonia from induced pluripotent stem cells in culture. Nat Protoc 2020;15:1560–1583. 32231324 10.1038/s41596-020-0297-5

[hoaf056-B89] Yokobayashi S , Okita K, Nakagawa M, Nakamura T, Yabuta Y, Yamamoto T, Saitou M. Clonal variation of human induced pluripotent stem cells for induction into the germ cell fate. Biol Reprod 2017;96:1154–1166. 28453617 10.1093/biolre/iox038

[hoaf056-B90] Yoshino T , Suzuki T, Nagamatsu G, Yabukami H, Ikegaya M, Kishima M, Kita H, Imamura T, Nakashima K, Nishinakamura R et al Generation of ovarian follicles from mouse pluripotent stem cells. Science 2021;373:eabe0237. 34437124 10.1126/science.abe0237

[hoaf056-B91] Yu X , Wang N, Wang X, Ren H, Zhang Y, Zhang Y, Qiu Y, Wang H, Wang G, Pei X et al Oocyte arrested at metaphase II stage were derived from human pluripotent stem cells in vitro. Stem Cell Rev Rep 2023;19:1067–1081. 36735215 10.1007/s12015-023-10511-7 PMC10185642

[hoaf056-B92] Zhou Q , Wang M, Yuan Y, Wang X, Fu R, Wan H, Xie M, Liu M, Guo X, Zheng Y et al Complete meiosis from embryonic stem cell-derived germ cells in vitro. Cell Stem Cell 2016;18:330–340. 26923202 10.1016/j.stem.2016.01.017

[hoaf056-B93] Zimmerman KD , Espeland MA, Langefeld CD. A practical solution to pseudoreplication bias in single-cell studies. Nat Commun 2021;12:738. 33531494 10.1038/s41467-021-21038-1 PMC7854630

[hoaf056-B94] Zuo Q , Jin J, Jin K, Sun C, Song J, Zhang Y, Chen G, Li B. Distinct roles of retinoic acid and BMP4 pathways in the formation of chicken primordial germ cells and spermatogonial stem cells. Food Funct 2019;10:7152–7163. 31596288 10.1039/c9fo01485c

